# Untargeted metabolomics identified kynurenine as a predictive prognostic biomarker in acute myocardial infarction

**DOI:** 10.3389/fimmu.2022.950441

**Published:** 2022-11-02

**Authors:** Xiaolin Zhang, Yi Cai, Xu Su, Quanmin Jing, Haiwei Liu, Kun Na, Miaohan Qiu, Xiaoxiang Tian, Dan Liu, Tianxiao Wu, Chenghui Yan, Yaling Han

**Affiliations:** ^1^ Second Affiliated Hospital of Dalian Medical University, Dalian, China; ^2^ Cardiovascular Research Institute and Department of Cardiology, The General Hospital of Northern Theater Command, Shenyang, China; ^3^ Key Laboratory of Structure-Based Drug Design and Discovery, Ministry of Education, School of Pharmaceutical Engineering, Shenyang Pharmaceutical University, Shenyang, China

**Keywords:** ST-acute myocardial infarction, Kynurenine, metabolites, prognosis, biomarker, indoleamine-pyrrole 2,3-dioxygenase

## Abstract

**Objective:**

The occurrence of cardiovascular adverse events in the first year after ST-acute myocardial infarction (STEMI) remains high; therefore, identification of patients with poor prognosis is essential for early intervention. This study aimed to evaluate the prognostic value of metabolomics-based biomarkers in STEMI patients and explore their functional mechanisms.

**Methods:**

Metabolite profiling was performed using nuclear magnetic resonance. The plasma concentration of Kynurenine (Kyn) was measured using ultraperformance liquid chromatography/electrospray ionization quadruple time-of-flight mass spectrometry. Major adverse cardiac and cerebral events were assessed for 1 year. A functional metabolomics strategy was proposed for investigating the role of Kyn in both vitro and vivo models.

**Results:**

The adjusted hazard ratios in STEMI patients for Kyn in the 4^th^ quartile 7.12(5.71-10.82) was significantly higher than that in the 3^rd^ quartile 3.03(2.62-3.74), 2^nd^ quartile 1.86(1.70-2.03), and 1^st^ quartile 1.20(0.93-1.39).The incidence of MACCE was significantly different among Kyn quartiles and the highest incidence of MACCE was observed in the 4th quartile when compared with the 1st quartile (9.84% vs.2.85%, P<0.001).Immunofluorescence staining indicated that indoleamine-pyrrole 2,3-dioxygenase (IDO1) was located in the CD68 positive staining area of thrombi from STEMI patients and Kyn was induced in the early phase after myocardial infarction. Kyn could trigger inflammation and oxidative stress of macrophage cells by activation of the Sirt3-acSOD2/IL-1β signaling pathway *in vitro*.

**Conclusions:**

Plasma Kyn levels were positively associated with the occurrence of STEMI. Kyn could induce macrophage cells inflammation and oxidative stress by activating the Sirt3-acSOD2/IL-1β pathway following myocardial ischemia injury. Kyn could be a robust biomarker for STEMI prognosis and reduction of Kyn could be beneficial in STEMI patients.

## Graphical Abstract

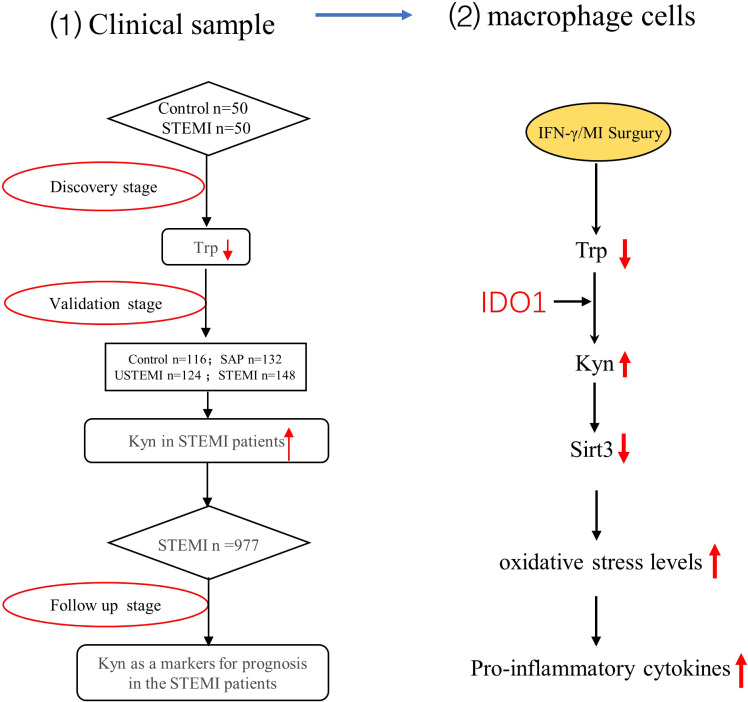

## Highlights

### What is new?

- A total of 15 kinds of metabolites markers were relate to the plasma of STEMI patients with time of onset less than 3h compared with the control subjects by ^1^H-NMR.
- This was the first study to assess the prognostic value of Kyn in STEMI patients.
- Kyn may act as a signaling molecule to activate the Sirt3-acSOD2/IL1β pathway leaded to macrophage cells inflammation and oxidative stress after myocardial ischemia injury.

### What are the clinical implications?

- Plasma Kyn concentration in STEMI patients is high and Kyn may act as a potential metabolic marker for the prognostic analysis of STEMI patients.
- Kyn may activate the Sirt3-acSOD2/IL1β pathway leaded to macrophage cells inflammation and oxidative stress. Therefore, inhibiting Kyn-related pathways such as reducing the plasma Kyn concentration or inhibiting Kyn-induced macrophage cells inflammation and oxidative stress may be used as potential therapeutic targets for STEMI patients.

## Introduction

The prevalence of ST-segment elevated myocardial infarction (STEMI) in the general population is rising and the mortality rates associated with its plaque rupture are high (1–3). The pathogenesis of STEMI is associated with the infiltration of inflammatory cells, cardiomyocyte apoptosis and fibrosis (4, 5). Improvements in primary treatment decrease the risk of cardiovascular events, however, this risk remains high during the first year after myocardial infarction (MI) (6–8). Therefore, the identification of patients with poor prognoses could help in optimizing therapy and outcomes. The available biomarkers and scoring systems for predicting cardiovascular adverse events in STMEI patients are being improved, however, predicting the risk of cardiovascular event outcomes during the first year after MI remains limited. Therefore, it is essential to optimize comprehensive approaches for prognosis assessment. This study aimed to evaluate the prognostic value of metabolomics-based biomarkers with patients in STEMI and explore their functional mechanisms.

Comprehensive metabolomics is an emerging tool in biology that would enable a better understanding of the pathogenesis associated with STEMI (8, 9). Characterization of metabolic markers and identification of regulatory enzymes will help identify new targets and design pharmacological interventions to improve the prognosis of patients with STEMI.

Recently, emerging studies have made comprehensive metabolomics assessments to identify biomarkers for the prognosis of STEMI (10). Ultraperformance liquid chromatography/electrospray ionization/quadruple time-of-flight mass spectrometry (UPLC/Q-TOF) has good reliability and reproducibility and is suitable for the sensitive detection of small molecule metabolites (10, 11). In our study we found the plasma concentration of Kynurenine (Kyn) was increased in patients with STEMI using UPLC/Q-TOF analysis.

Kyn, the major metabolite of tryptophan (Trp), is involved in several fundamental biological processes. Indoleamine 2, 3-dioxygenase, a rate-limiting enzyme that catalyzes the degradation of Trp to Kyn is an important regulator of several pathological conditions (12, 13). A previous clinical study showed that circulating Kyn, a major IDO-related metabolites was associated with cardiovascular risk factors and with worse cardiovascular outcomes in stable coronary artery disease patients (13–16). In contrast the functional analysis showed that either protective, or deleterious effects of inhibitors involving Trp to Kyn conversion have been reported. Sarvenaz Metghalchi et al. reported that decreasing the Trp to Kyn conversion protected against atherosclerosis (12, 17). In contrast, Ketelhuth, K. et al. reported that inhibiting endogenous Kyn production leads to increased vascular inflammation and aggravated atherosclerosis (16). Together, these findings suggested the complexity of Trp degradation in the Kyn metabolism pathway. To clarify the role of Kyn in the occurrence of acute myocardial infarction (AMI), we evaluated the power of plasma Kyn levels in a large series of patients with STEMI and aimed to characterize the role of Kyn in myocardial injury. Based on these observations, a functional metabolomics strategy was used to identify the possible role of Kyn in AMI progression.

## Materials and methods

### Study design and participants

In order to clarify the characteristics of plasma metabolism in STEMI patients, 50 consecutive STEMI patients presenting with acute chest pain to the emergency department and 50 control subjects were recruited from January 2014 to June 2014 for the ^1^H-NMR analysis. To verify the results of ^1^NMR, 116 control subjects, 132 stable angina pectoris (SAP) patients,124 non-ST-segment elevated myocardial infarction (NSTEMI) patients and 148 STEMI patients were recruited from April 2014 to October 2014 to evaluate the Trp and its metabolites concentration using the LC-MS/MS. The baseline characteristics and clinical parameters were shown in **Table 1**.

**Table 1:** Baseline characteristics of the recruited subjects.

	Overall (n=520)	Control (n=116)	SAP (n=132)	NSTEMI (n=124)	STEMI (n=148)	P value
Age, year	60.24 (10.99)	59.82 (10.86)	62.27 (9.17)	59.26 (10.69)	59.59 (12.58)	0.102
Male	372 (71.54%)	69 (59.48%)	88 (66.67%)	97 (78.23%)	118 (79.73%)	0.001
BMI, kg/m2	25.53 (2.98)	25.44 (3.20)	25.51 (2.70)	26.38 (3.31)	24.92 (2.58)	0.001
HR, per minute	78.33 (13.41)	75.04 (10.87)	78.67 (13.01)	78.10 (11.77)	80.80 (16.15)	0.007
Current smoker	294 (56.54%)	57 (49.41%)	75 (56.81%)	66 (53.22%)	96 (64.86%)	0.062
Hypertension	287 (55.19%)	55 (47.41%)	72 (54.55%)	67 (54.03%)	93 (62.83%)	0.093
Diabetes	132 (25.38%)	25 (21.55%)	31 (23.48%)	35 (28.23%)	41 (27.70%)	0.552
TG, mmol/L	1.89 (1.59)	1.65 (0.82)	2.15 (1.99)	1.93 (1.12)	1.79 (1.91)	0.071
HDL-C,mmol/L	1.05 (0.32)	0.94 (0.20)	0.93 (0.21)	1.21 (0.47)	1.11 (0.23)	<0.001
LDL-C, mmol/L	2.50 (0.94)	2.16 (0.95)	2.40 (0.87)	2.25 (0.83)	3.05 (0.85)	<0.001
Gl ucose, mmol/L	6.88 (2.33)	6.02 (1.86)	6.46 (2.05)	7.08 (2.46)	7.76 (2.46)	<0.001
WBC, 10^9/L	8.64 (3.21)	7.25 (1.94)	7.03 (1.64)	8.45 (2.44)	11.31 (3.85)	<0.001
hsCRP, mg/dL	2.26 (1.11, 3.78)	2.03 (1.08, 3.30)	2.37 (1.44, 3.80)	1.93 (0.98, 3.33)	2.87 (1.11, 6.47)	0.019
cTnT, μg/L	0.03 (0.01, 0.29)	0.01 (0.00, 0.01)	0.01 (0.01, 0.01)	0.20 (0.11, 0.36)	0.53 (0.12, 2.14)	<0.001
Creatine kinase, MB, U/L	14.50(10.00, 44.50)	11.00 (9.00, 13.00)	11.00 (9.00, 14.00)	42.50 (15.00, 67.50)	39.00 (15.00, 109.75)	<0.001
TRP(μmol/L)	47.25 (37.01, 55.37)	45.42 (37.12, 51.03)	46.91 (40.51,50.66)	50.95 (37.19, 59.74)	49.03 (34.41, 64.66)	0.012
Kyn(μmol/L)	1.46 (1.21,2.32)	1.21 (1.05, 1.34)	1.23 (1.07, 1.58)	1.88 (1.45, 2.15)	3.24 (1.51, 5.26)	<0.001

TG, triglyceride; TC, total cholesterol; LDL-C, low-density lipoprotein; HDL-C, high density lipoprotein; BUN, Blood Urea Nitrogen; Crea, creatinine; GLU, blood glucose; WBC, white blood cell; hs-CRP, high-sensitivity C-reactive protein; hscTnT, high-sensitivity troponin T; CK-MB, creatine kinase isoenzymes. Data presented are the means ± SD or numbers of patients (percentage).

### Follow-up and end points

As a whole, we further analyzed the prognostic value of Kyn in 977 STEMI patients recruited from January 2015 to April 2017. The study protocol was approved by the General Hospital of Northern Theater Command Ethics Committee, and written informed consent was obtained from all subjects.

### Metabolomics analysis based on ^1^H NMR

300 μL plasma samples were mixed with methanol in a 1:2 ratio (v/v), vortexed, and incubated at −20°C for 20 min. The mixtures were centrifuged at 11,000 rpm for 30 min to pellet proteins. All the samples were centrifuged at 12,000 rpm for 10 min before transferred into the NMR tubes for the detection.

### Targeted analysis for tryptophan and its metabolites by LC-MS/MS

Liquid chromatographic separation for processed plasma was achieved on a UPLC HSS C_18_ column (100mm×2.1mm,1.7μm) using a UPLC/Q-TOF (Waters Corp., Milford, MA, USA).

### Biomarker measurement

IDO1 concentration in the plasma of the STEMI patients and control subjects was determined using an enzyme-linked immunosorbent assay (ELISA) according to the manufacturer’s instructions (Novus NBP2-62765, American).

### Human thrombi tissues in STEMI patients

The coronary thrombus tissues from STEMI patients were obtained by aspiration catheter.

### Cell preparation and stimulation

RAW264.7(m Macrophage cell) and HL-1(m myocardial cell) were from American Type Culture Collection. H endothelial cell were cultured of human umbilical vein endothelial cells. Human umbilical vein endothelial cells were cultured primarily in vitro. Human vascular smooth muscle cells (hVSMCs) were grown in DMEM to 70% to 80% confluence. Cells were used between passages 3 and 8, and grown to 70% to 80% confluence before being used.

### *In Vivo* siSirt3 and pcDNA3.1-Sirt3 delivery

The pre-designed siRNA delivery was carried out according to the RiboBio’s *in vivo* RNAi protocol. The CDS region of mouse Sirt3 gene was inserted in pcDNA3.1 vector which were synthesized by GENEWIZ (Suzhou, China).

### Immunohistochemical analyses and immunofluorescence staining

Immunohistochemical analyses were performed using paraffin-embedded tissue sections. All tissue samples were characterized using hematoxylin and eosin (H&E) staining before further immunostaining. The sections were incubated with primary anti-Sirt3, anti-IDO1 and anti-CD68 antibodies at 4°C overnight. After the visualization with 3,3’-diaminobenzidine, the sections were counterstained with hematoxylin. In the immunofluorescence staining the primary antibodies were applied at 4°C overnight, and bound antibodies were detected *via* goat anti-rabbit Alexa Fluor 488 and donkey anti-mouse Alexa Fluor 555 (Invitrogen, Thermo Fisher Scientific, and Carlsbad, CA, USA).

### MitoSOX red stain

To examine mitochondrial reactive oxygen species (ROS) levels, cells were loaded with 5 μM MitoSOX Red for 10 min at 37°C, which was a mitochondrial superoxide indicator.

### Detection of superoxide dismutase

Measurement of superoxide dismutase (SOD) level was performed using commercial assay Kits (Solarbio Chemical Company, China) as described by the manufacturer.

### Determination of tryptophan,3-Hydroxy-DL-kynurenine, kynurenic acid, xanthurenic acid, and quinolinic acid

Aliquots of 200 μL methanol and 10 μL IS (Trp-d3;10 μg/ml) were added to the sample to precipitate protein. Then, 200 μL of each supernatant was allowed to dry out under a nitrogen stream at 35°C. Each residue was redissolved in 50 μL acetonitrile-water (50:50 v/v) and passed through a 0.22 mm microporous membrane filter. Target analysis for Trp and its metabolites by LC-MS/MS was as described previously.

### Molecular docking study

The Sirt3 crystal structure which was downloaded from protein data bank (https://www.rcsb.org/) was processed with the Protein Preparation Wizard in the Schrçdinger suite.

### Isolation of human peripheral blood monocytes

We withdrew samples of peripheral blood from STEMI patients and control volunteers, and then isolated PBMCs using the Ficoll-density-gradient separation as previously described. We lysed purified PBMCs in a lysis buffer and a protease inhibitor cocktail, and stored them at -80°C until use.

### Statistical analysis

Categorical variables were reported as counts and percentages, and between-group differences were assessed with chi-square or Fisher’s exact test. Continuous variables were presented as the mean ± SD and were compared with one-way analysis of variance. We examined patients’characteristics, treatments, tests, procedures, and crude rate of outcomes across quartiles of Kyn concentration using the Cochran-Armitage trend test for the trend of binary variables, and the Mann-Kendall trend test for trends of continuous variables. Survival curves for time-to-event variables were compared by the log-rank test. Receiver operating characteristic curve for concentration Kyn was constructed to assess the predictive accuracy for 1-year MACCE. The non-linear association was further evaluated by using restricted cubic splines between concentration Kyn and 1-year MACCE. The logistic regression was used to investigate the independent predictors for MACCE at 1 year. Statistical analysis was performed using SPSS 23.0 (IBM SPSS Inc., Chicago, IL, USA) and R-3.6.3 (R Core Team, Vienna, Austria). A two-tailed P< 0.05 was considered statistically significant.

## Result

### Plasma Kyn levels were elevated in patients with STEMI

A total of 50 STEMI patients with time of onset less than 3h and 50 control subjects were enrolled in the discovery stage. Untargeted metabolomics was performed by ^1^H-NMR analysis, and the baseline data of the selected participates were shown in **Supplemental Table 1**. Plasma concentrations of 15 metabolites were significantly different between STEMI patients and control subjects. Among them, the Trp metabolite pathway was the most downregulated in STEMI patients (**Supplemental Table 3**). We verified this using targeted mass spectrometry in the validation stage; data were available for 520 individuals including 148 STEMI patients, 124 NSTEMI patients,132 SAP patients and 116 controls participants. The baseline characteristics and clinical parameters were recorded in **Table 1**. The STEMI patients with a higher prevalence of hypertension compared to that in the other groups, were probably smokers and had the highest white blood cell counts among the four groups. The plasma Kyn levels were the highest in STEMI patients (3.24 μmol/ml), compared to that in the control subjects (1.21 μmol/ml), SAP patients (1.23 μmol/ml), and NSTEMI patients (1.88 μmol/ml) (**Table 1**). Other metabolites of Trp downstream did not change significantly after myocardial infarction; they even decreased, suggesting a minor role for those metabolites in the occurrence of STEMI.

### The Kyn levels were higher in STEMI patients who developed MACCE

**Figure 1:**
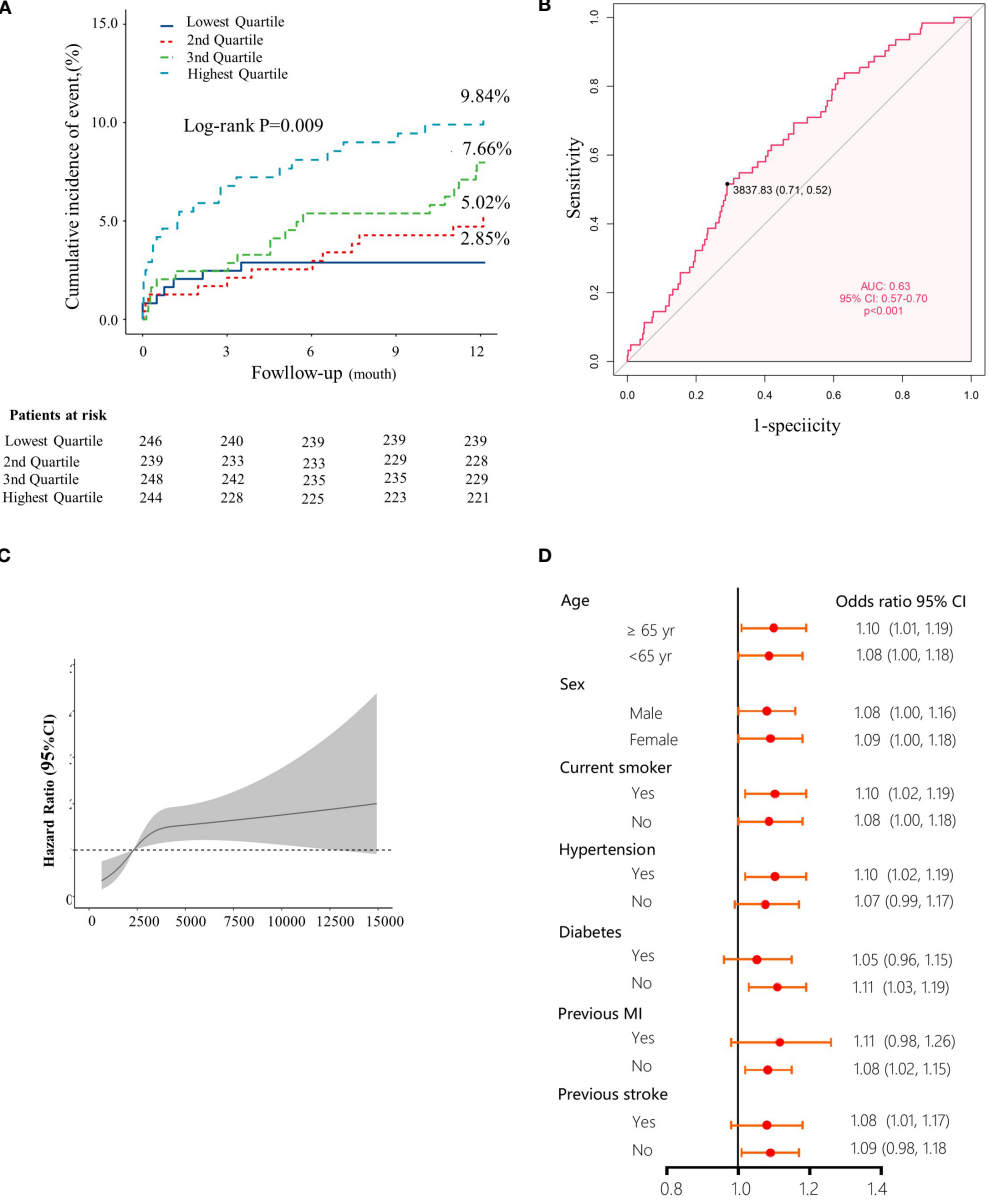
High levels of Kyn as a prognostic indicator in STEMI patients. **(A)** Kaplan-Meier plots indicated the association between quartile levels of Kyn and major adverse cardiovascular events, the numbers of STEMI patients in the different quartile groups were included in each graph. **(B)** Receiver operating characteristic curves for the patients with STEMI vs. control subjects for Kyn. **(C)** Restricted cubic spline modeling indicated the relationship between the plasma Kyn levels and the risk of MACCE. The solid line indicated the hazard ratios and the shaded areas, the 95% confidence intervals. **(D)** Forest plots showed that Kyn levels were associated with the prognosis in STEMI patients of subgroups according the STEMI prognostic analysis cohort data, after adjusting for per-standard deviation odds ratio (95% confidence interval) for the plasma.

To the best of our knowledge, this is the first study to evaluate the effect of plasma Kyn concentration on the clinical outcomes of STEMI patients. To investigate the prognostic value of Kyn, 977 STEMI patients were followed up for 1 year. 62 (6.35%) patients developed major adverse cardiac and cerebral events (MACCE). Quartiles of Kyn in relation to demographic characteristics, medication data, and coronary intervention results were included in **Table 2**. Higher levels of plasma Kyn were associated with higher proportions of diabetes, higher smoking, previous MI, previous stroke, and higher cTnT and NT-proBNP levels. The number of events during the 1-year follow-up was included in **Table 3**. In addition, increased level of plasma Kyn was associated with increased risk of one-year MACCEs (2.85% vs. 5.02% vs. 7.66% vs. 9.84% for patients in 1^st^, 2^nd^, 3^rd^ and 4^th^ quartile, respectively, P for trend = 0.001) (**Figure 1A**). The Kaplan–Meier estimates of the primary outcome across quartiles of Kyn concentration was presented in **Figure 1B**. The levels of plasma Kyn was associated with all-cause death (2.06% vs. 2.98% vs. 3.64% vs. 5.81%, P for trend = 0.027) and rehospitalization for heart failure (0.00% vs. 0.59% vs. 3.28% vs. 2.37%, P for trend = 0.024) (**Table 3**). The Kyn concentration showed a moderate prediction value for 1-year MACCE in the ROC analysis with an AUC of 0.63 (95% CI 0.57-0.70) (**Figure 1C**). A nonlinear pattern was observed between Kyn levels and 1-year MACCE events with consistently increased risk (**Figure 1D**). Sensitivity analysis revealed that the Kyn level was consistently associated with increased risk of 1-year MACCE.

**Table 2:** Baseline characteristics of cohort 4 STEMI patients as stratified by baseline Kyn quartiles.

		Plasma Kyn levels on admission				
	Overall(n=977)	Lowest Quartile(n=246)	2nd Quartile(n=239)	3rd Quartile(n=248)	Highest Quartile(n=244)	P for trend
Age (years)	59.30 ± 12.28	59.93 ± 12.48	59.65 ± 12.19	58.10 ± 11.90	59.52 ± 12.53	0.427
Male	782 (80.0%)	200 (81.3%)	193 (80.8%)	200 (80.6%)	189 (77.5%)	0.310
Smoking history	604 (62.1%)	151 (62.1%)	148 (61.9%)	149 (60.1%)	156 64.2%)	0.759
Alcohol history	230 (31.1%)	45 (26.6%)	59 (34.1%)	58 (31.5%)	68 (31.8%)	0.427
Hypertension	557 (57.1%)	152 (62.0%)	133 (55.6%)	142 (57.3%)	130 (53.3%)	0.081
Diabetes	306 (31.4%)	65 (26.5%)	72 (30.3%)	75 (30.2%)	94 (38.5%)	0.007
Previous MI	89 (9.13%)	24 (9.84%)	13 (5.44%)	22 (8.87%)	30 (12.3%)	0.193
Previous stroke	156 (16.0%)	38 (15.6%)	35 (14.6%)	34 (13.7%)	49 (20.1%)	0.233
Family history of AMI	19 (2.57%)	5 (2.96%)	8 (4.62%)	4 (2.17%)	2 (0.93%)	0.085
Anemia	208 (21.3%)	54 (22.0%)	49 (20.5%)	44 (17.7%)	61 (25.0%)	0.592
eGFR, mL/min/1.73 m^2^	101 (33.4)	101 (31.9)	102 (31.4)	102 (33.5)	100 (36.5)	0.757
LVEF (%)	53.8 (9.32)	53.2 (9.72)	54.9 (9.04)	53.0 (9.59)	54.4 (8.82)	0.503
**Laboratory test**						
RBC, 10 ^12^/L	4.63 (0.65)	4.63 (0.62)	4.60 (0.66)	4.64 (0.64)	4.67 (0.70)	0.383
Hemoglobin, g/dL	140 (17.9)	139 (16.4)	140 (21.8)	141 (17.2)	139 (16.0)	0.894
TC, mmol/L	5.01 (1.22)	5.02 (1.12)	4.98 (1.30)	5.02 (1.27)	5.01 (1.20)	0.979
TG, mmol/L	1.86 (1.79)	1.86 (1.88)	1.89 (1.82)	1.87 (1.88)	1.81 (1.57)	0.735
LDL-C, mmol/L	3.03 (0.79)	3.00 (0.75)	3.04 (0.80)	2.99 (0.81)	3.10 (0.81)	0.242
HDL-C, mmol/L	1.05 (0.23)	1.02 (0.21)	1.05 (0.24)	1.07 (0.23)	1.05 (0.21)	0.123
BUN, mmol/L	5.58 (1.87)	5.47 (1.77)	5.42 (1.73)	5.55 (1.89)	5.81 (2.01)	0.049
CCr, umol/L	76.6 (26.3)	75.8 (21.9)	75.0 (23.0)	77.8 (31.3)	77.9 (27.9)	0.239
NT-proBNP, pg/ml	1897 (3224)	1725 (2248)	1547 (2233)	1792 (2746)	2501 (4809)	0.006
cTnT, μg/L	3.82 (1.80, 6.99)	3.62 (1.58, 7.00)	3.62 (1.90, 6.14)	3.37 (1.51, 6.20)	4.70 (2.28, 7.80)	0.029
Creatine kinase, MB, U/L	188.00 (106.00, 316.00)	178.00 (95.25, 325.75)	183.00 (96.00, 313.50)	201.50 (104.50, 306.25)	197.5 0 (121.75, 337.25)	0.317
**Lesion characteristics**						
Target vessel location						
Left main artery	19 (1.98%)	2 (0.82%)	6 (2.55%)	5 (2.05%)	6 (2.51%)	0.250
Left anterior descending artery	600 (62.4%)	143 (58.6%)	145 (61.7%)	153 (62.7%)	159 (66.5%)	0.076
Left circumflex artery	237 (24.6%)	59 (24.2%)	56 (23.8%)	55 (22.5%)	67 (28.0%)	0.414
Right coronary artery	441 (45.8%)	113 (46.3%)	111 (47.2%)	110 (45.1%)	107 (44.8%)	0.639
Transradial approach	826 (85.9%)	207 (84.8%)	199 (84.7%)	209 (85.7%)	211 (88.3%)	0.262
**Medications at discharge**						
Aspirin	964 (98.9%)	240 (98.0%)	236 (99.2%)	246 (99.2%)	242 (99.2%)	0.219
P2Y12 Receptor Inhibitor						
Clopidogrel	617 (63.3%)	156 (63.7%)	156 (65.5%)	161 (64.9%)	144 (59.0%)	0.293
Ticagrelor	358 (36.7%)	88 (35.9%)	83 (34.9%)	87 (35.1%)	100 (41.0%)	0.267
β-blockers	780 (79.8%)	198 (80.5%)	191 (79.9%)	185 (74.6%)	206 (84.4%)	0.579
ACEI/ARB	737 (75.4%)	181 (73.6%)	179 (74.9%)	194 (78.2%)	183 (75.0%)	0.534
Statins	946 (96.8%)	237 (96.3%)	234 (97.9%)	242 (97.6%)	233 (95.5%)	0.574
**Plasma biomarkers related to the kynurenine pathway**						
Kyn		1.20(0.93, 1.39)	1.86(1.70, 2.03)	3.03(2.62,3.74)	7.12(5.71, 10.82)	<0.001

Data are Median (Lowest Quartile, Highest Quartile) or n (%). PCI, percutaneous coronary intervention; LAD, left anterior descending; LCX, left circumflex artery; RCA, right coronary artery; LM, left main; LVEF, left ventricular ejection fraction; ACE, angiotensin-converting enzyme. Other abbreviations as in **Table 1**.

**Table 3:** One-year clinical outcomes in cohort 4 STEMI patients according to peak Kyn quartiles.

		Plasma Kyn levels on admission				
	Overall(n=977)	Lowest Quartile(n=246)	2ndQuartile(n=239)	3rdQuartile(n=248)	HighestQuartile(n=244)	P for trend
MACE*	62 (6.35%)	7 (2.85%)	12 (5.02%)	19 (7.66%)	24 (9.84%)	0.001
All-cause mortality*	35 (3.62%)	5 (2.06%)	7 (2.98%)	9 (3.64%)	14 (5.81%)	0.027
Ischaemic stroke	10 (1.04%)	1 (0.41%)	3 (1.28%)	1 (0.40%)	5 (2.07%)	0.158
MI	4 (0.41%)	1 (0.41%)	1 (0.43%)	1 (0.40%)	1 (0.41%)	0.996
Rehospitalization for heart failure	12 (1.65%)	0 (0.00%)	1 (0.59%)	6 (3.28%)	5 (2.37%)	0.024

MACCE, major adverse cardiac and cerebral events.

Univariate and multivariate logistic regression analyses results for MACCE at 1-year follow up were included in **Table 4**. In the multivariate analysis, age, history of stroke, LVEF, NT-proBNP, and plasma Kyn levels were independent predictors of MACCE. Plasma Kyn level was a good predictor for MACCE either as a continuous variable (OR: 1.09, 95%CI 1.03-1.15) or as an ordinal variable, compared to that in the lowest quartile as the reference; the ORs of quartiles 2, 3, 4 were 1.80 (95% CI 0.70-4.67), 2.83 (95% CI 1.17-6.87) and 3.72 (95% CI 1.57-8.82).

**Table 4:** Logistic Regression Analysis for MACE in Patients with STEMI.

	Simple Regression		Multiple Regression	
	OR (95% CI)	P-value	OR (95% CI)	P-value
Age (years)	1.04 (1.02, 1.07)	<0.001	1.03 (1.01, 1.06)	0.019
Male	0.42 (0.25, 0.73)	0.002		
Current smoker	0.71 (0.42, 1.20)	0.202		
Hypertension	1.04 (0.62, 1.76)	0.870		
Diabetes	1.52 (0.90, 2.57)	0.119		
Previous MI	1.07 (0.45, 2.56)	0.877		
Previous stroke	2.15 (1.20, 3.88)	0.011	2.35 (1.22, 4.50)	0.010
LVEF (%)	0.96 (0.93, 0.98)	0.001	0.97 (0.94, 1.00)	0.047
TC, mmol/L	0.89 (0.70, 1.14)	0.357		
TG, mmol/L	0.85 (0.65, 1.11)	0.228		
LDL-C, mmol/L	1.28 (0.91, 1.81)	0.151		
HDL-C, mmol/L	1.23 (0.36, 4.18)	0.737		
cTnT, μg/L	1.03 (0.95, 1.12)	0.459		
NT-proBNP, pg/ml	1.0001 (1.0001, 1.0002)	<0.001	1.0001 (1.0000, 1.0001)	0.034
KYN/1000 (continuous variable)	1.09 (1.03, 1.15)	0.003		
KYN (classification variable)				
2nd Quartile	1.80 (0.70, 4.67)	0.223	3.14 (0.97, 10.13	0.055
3rd Quartile	2.83 (1.17, 6.87)	0.021	4.43 (1.44, 13.63)	0.009
Highest Quartile	3.72 (1.57, 8.82)	0.003	5.31 (1.76, 16.01)	0.003

Abbreviations per **Table 2**.

### IDO1 expression in thrombi tissues

IDO1, the rate-limiting enzyme of the Trp pathway, regulates the conversion of the essential amino acid Trp into several downstream metabolites, collectively referred to as Kyn.

**Figure 2:**
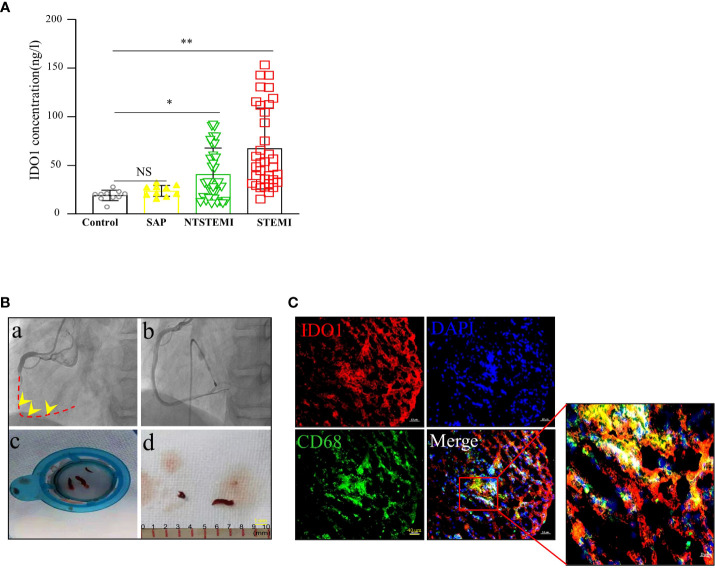
High IDO1 and Kyn expression in thrombi of coronary arteries. **(A)** Distribution of IDO1 plasma level in control subjects(n=10), SAP patients(n=10), NTSTEMI patients(n=34) and STEMI patients(n=35), NS, not significant, *P<0.05 vs con subjects, **P<0.01 vs con subjects. **(B)** Coronary artery thrombi were obtained from the STEMI patients presenting to the cardiac catheterization laboratory. **(a)** Angiogram demonstrating thrombotic occlusion (arrow) of the right coronary artery in the STEMI patient, dashed line indicated the occluded vessels. **(b)** Final angiogram showed a widely patent of the right coronary artery with no significant luminal narrowing. **(c, d)** Thrombectomy catheter aspirate after passage through filter showing coronary artery thrombi (arrow) (scale bar, 1mm). **(C)** Representative immunofluorescence staining for CD68 and IDO1 in the coronary artery thrombi of STEMI patients. Immunofluorescence image showing CD68^+^(red) macrophages and IDO1 (green) expression; merged image was included. Nuclei was counterstained with DAPI. Co-localization of IDO1 and CD68^+^ macrophages was depicted in the overlay. The square indicated the macrophage cells expressing IDO1(scale bar, 40 μm).

The levels of plasma Kyn were verified based on the IDO1 activity during the inflammatory immune responses. The plasma IDO1 activity was enhanced in STEMI patients (67.45 ± 41.59 ng/ml) compared to that in the control subjects (19.24 ± 5.34 ng/ml), SAP patients (23.82 ± 5.61 ng/ml) and NSTEMI patients (40.80 ± 27.64 ng/ml) (**Figure 2A**).

STEMI is mostly caused by atherosclerotic plaque rupture and occlusive thrombus formation. To examine the pathophysiological relevance of increased IDO1 expression in thrombi tissues, we obtained coronary artery thrombi from STEMI patients who presented to the cardiac catheterization laboratory. Angiography demonstrated that the thrombotic occlusion of the right branch coronary artery which was treated with aspiration thrombectomy followed by balloon angioplasty and stent deployment (**Figure 2B**), resulted in a widely patent right branch coronary artery with no significant luminal narrowing. The thrombectomy catheter retrieved multiple coronary artery thrombi that were analyzed for the presence of CD68^+^ macrophages and IDO1 expression using immunofluorescence microscopy. CD68^+^ macrophages were abundant and they co-localized with IDO1 expression in the human coronary artery thrombus (**Figure 2C**).

### IDO1 was upregulated in cardiac macrophage cells following MI

**Figure 3:**
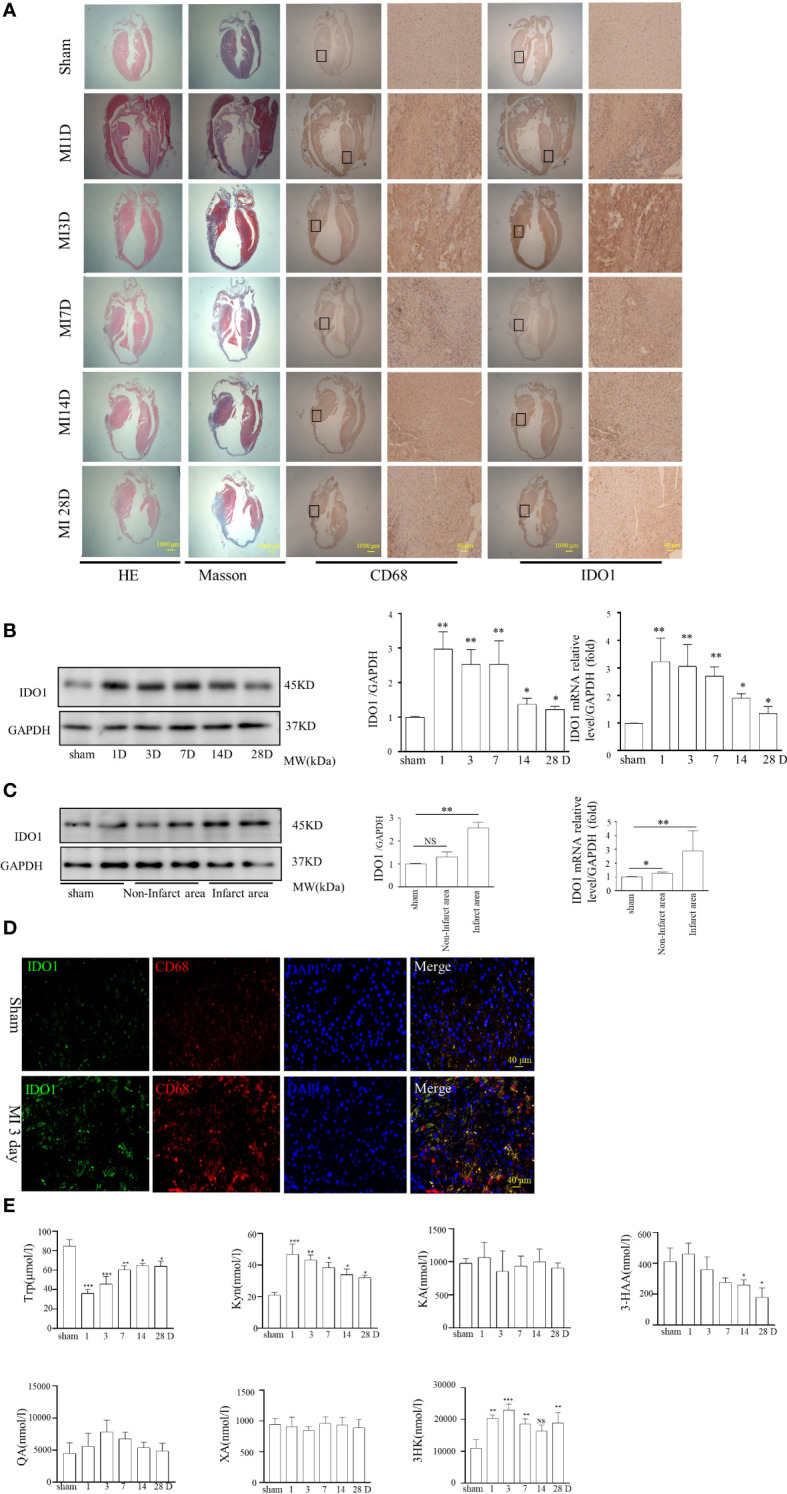
IDO1 expression in the cardiac macrophage cells and the plasma of mice remodeling after MI. **(A)** Induction of MI by the permanent ligations of the left anterior descending artery (MI) at 1d, 3d, 7d, 14d, and 28d points or sham operations without ligation were performed on the experimental animals as described previously. Immunohistochemical analyses showed that IDO1 and CD68 were co-localized in the infarct and non-infarct area at different time points after MI, indicating that IDO1 was highly expressed in macrophage cells especially during 1−3 days after MI (scale bar, 1000 μm, scale bar, 40 μm). **(B)** IDO1 expression in heart was determined at different time points after MI using western blot analysis, NS, not significant, *P<0.05 vs sham group, **P<0.01 vs sham group, n=3. **(C)** IDO1 expression in the infarct and non-infarct area after MI was showed in the western blot analysis, *P<0.05 vs sham group, **P<0.01 vs sham group, n=3. **(D)** The colocalization of IDO1 with CD68 demonstrated with immunofluorescence in macrophage cells. **(E)** Distribution of Trp metabolites between the sham and MI group at different time points. Trp, tryptophan; Kyn, kynurenine; 3-HK, 3-hydroxykynurenine; KA, kynurenic acid; XA, xanthurenic acid; 3-HAA, 3-hydroxyanthranilic acid, *P<0.05 vs sham group, **P<0.01 vs sham group, n=3.

IDO1 expression was upregulated in macrophage cells of coronary artery thrombus tissues indicating that IDO1 was involved in the ischemia injury and impaired wound healing. Immunohistochemistry staining and western blot showed the expression of IDO1 was much higher in the infarct area than in the noninfarcted area at the 1,3,7,14 and 28 days after MI (**Figures 3A, B**). This trend was more evident in 1-3 day, which was confirmed by western blot and Real-Time PCR analysis (**Figure 3C**). Immunofluorescence staining showed that IDO1 (green) co-located with CD68 (red) macrophage cells (**Figure 3D**). Moreover the concentration of Kyn obviously increased at the 1,3,7,14 and 28 day after MI by UPLC/Q-TOF analysis, reached to the peak 1 to 3 day after the onset of MI, then gradually decreased (**Figure 3E**).

### The role of Sirt3-acSOD2/IL-1β pathway in Kyn-induced macrophage cells activation in inflammatory response and oxidative stress

**Figure 4:**
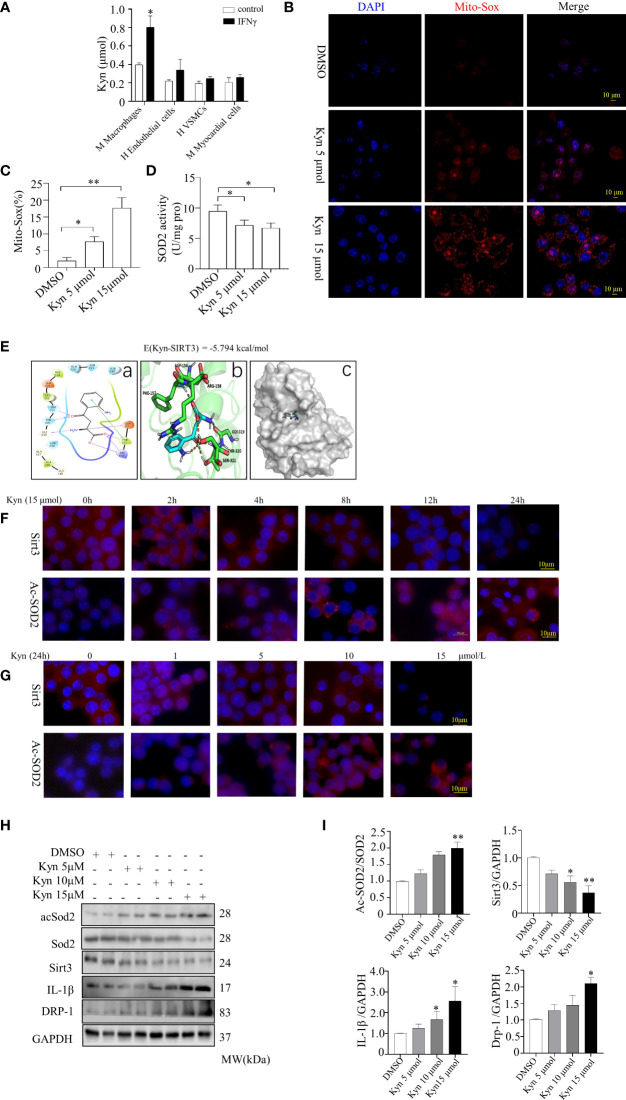
Kyn induced expression of inflammatory factors and increased the oxidative stress levels in macrophage cells and induced Sirt3. **(A)** Kyn expression were observed in the supernatant of macrophages, endothelial cells, VSMCs and myocardial cells stimulated with 20 ng/ml IFNγ for 24 h. **(B, C)** Mitochondria-derived ROS stimulated by Kyn after 24 h was detected using MitoSox-probe (Red) and the nuclei were stained with Hoechst stain (Blue), (scale bar, 10 μm),*P<0.05 vs DMSO, **P<0.01 vs DMSO, n=3. **(D)** SOD activity was determined using a detection kit. Kyn increased the mitochondrial oxidative stress activity in macrophage cells, *P<0.05 vs DMSO, n=3. **(E)** Interaction of Kyn with Sirt3, the AutoDock predicted binding modes of Kyn with Sirt3, in which the carboxyl group at the Kyn terminus formed salt-bonding interactions with Arg-158 and hydrogen-bonding interactions with Phe-157 and Arg-158. The benzoyl group and the protonated amino group formed a cross-linked hydrogen bond network with Gly-319, Thr-320 and Ser-321. The binding energy predicted by autodock was displayed. **(a)** Two dimensional structure diagram, **(b)** Three dimensional structure diagram, **(c)** Overall structure diagram. **(F, G)** Immunofluorescence staining showed that Kyn induced Sirt3 and acSOD2 expression in a dose- and time-dependent manner. **(F)** Macrophage cells were treated with 15µM Kyn according to the different time. **(G)** Macrophage cells were incubated according to the various concentration of Kyn for 24 h (scale bar, 10 μm). **(H, I)** Representative blots and quantification of acSOD2, Sirt3, IL-1β and DRP-1 protein expression of macrophage cells stimulated by 5,10 and 15 μmol Kyn differently. *P<0.05 vs DMSO, **P<0.01 vs DMSO, n=3.

Kyn levels were higher in the supernatant of macrophages (Raw264.7, mouse macrophage cells) compared with the endothelial cells (human endothelial cell), vascular smooth muscle cells (human VSMCs), and myocardial cells (mouse myocardial cell), following stimulation with 20 ng/ml IFNγ for 24 h (**Figure 4A**). The MitoSox-probe expression intensity indicated that the mitochondrial-derived ROS was obvious enhanced after Raw264.7 macrophage cells stimulated by Kyn (5 μmol and 15 μmol) for 24h (**Figures 4B–D**).

Docking experiments were performed to investigate the downstream targets of Kyn. The carboxyl group at the Kyn terminus formed the salt-bonding sites with Arg-158 and interacted with Phe-157 and Arg-158 of Sirt3 by hydrogen-bonding, and the binding energy was -5.794 kcal/mol. The benzoyl group and the protonated amino group formed a cross-linked hydrogen bond network with Gly-319, Thr-320 and Ser-321. Further stabilization was achieved through the π-π stacking (edge to face) interaction of the benzene ring with the Phe-157 which indicating that Kyn interacted with Sirt3 stably (**Figure 4E**).

Sirt3, a metabolic sensor, can use intracellular metabolites such as NAD+ and acetyl-CoA for modulating mitochondrial function to match nutrient supply which plays a crucial role in cardiovascular diseases. We tested whether Kyn could bind to Sirt3 and influence Sirt3 function. Sirt3 depletion led to superoxide dismutase-2 (SOD2) inactivation, this aggravated the mitochondrial oxidative stress response. DRP-1, a mitochondria fission protein, alleviates mitochondrial ROS in inflammatory responses. In order to assess the role of Kyn, we stimulated Raw264.7 macrophage cells with Kyn at different concentrations (from 5 to15 μmol/L) much higher than that detected in the plasma. The macrophage cells were also stimulated for 2 h, 4 h, 8 h, 12 h, or 24 h differently. Sirt3 was markedly downregulated and the expression of acetyl superoxide dismutase 2 (acSOD2) was induced following treatment with 15 μmol Kyn for 24 h (**Figures 4F, G**). Kyn upregulated IL-1β, DRP-1, and acSOD2 expression; it downregulated Sirt3, as observed using western blot analysis. Therefore, Kyn could increase oxidative stress and inflammatory response in Raw264.7 macrophage cells (**Figures 4H, I**).

### Supplementation with Kyn promoted macrophage cells inflammation *in vivo* and increased lesion size

**Figure 5:**
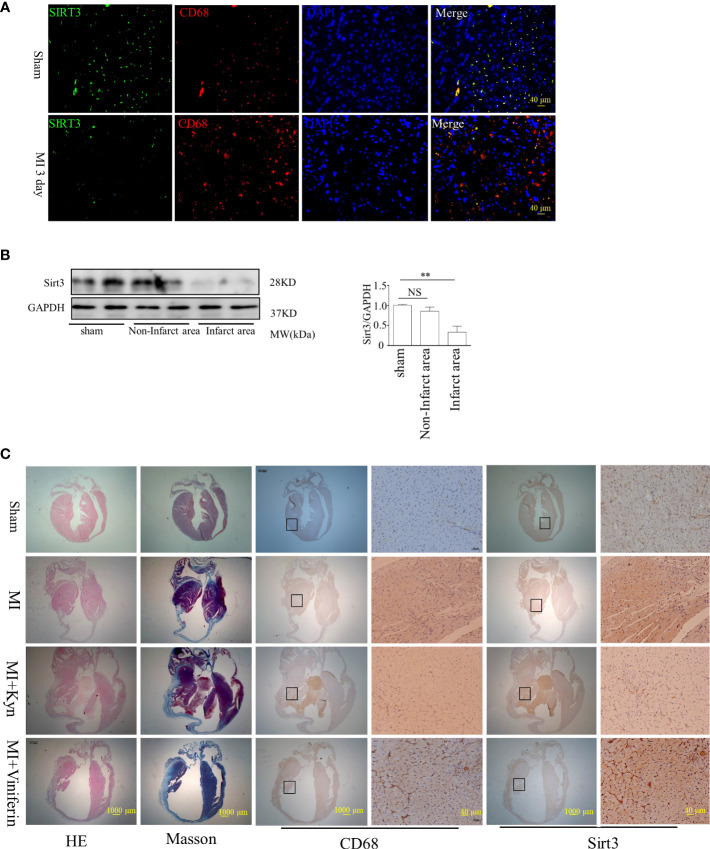
Kyn enhanced MI lesion size, and reversely Sirt3 activation alleviated the inflammatory response in the lesion area of MI. **(A)** Immunohistochemical analyses showed that Sirt3 and CD68 co-localized in the infarct area after MI, indicating that Sirt3 decreased in macrophages especially during the 1−3 days after MI, scale bar, 40 μm. **(B)** Sirt3 expression levels were analyzed in both infarct and non-infarct area after MI, n=3. NS, not significant, **P<0.01 vs sham. **(C)** Representative images showed H&E and Masson staining of myocardial tissue sections from control (sham) and MI mice subjected to Kyn (15μmol) treatment. Kyn supplementation deteriorated the cardiac function in MI mice *in vivo*, and the infiltration of CD68^+^ macrophages in the region of MI was aggravated. Treating mice with the Sirt3 activator can significantly reduce the area of MI and improve the cardiac function after MI; in addition, the infiltration of macrophages at the site of MI was reduced, indicating that the Sirt3 activator (Viniferin) can decrease the inflammatory response in the area of MI, scale bar, 10 μm, scale bars 40 μm.

Sirt3 expression decreased at 3 days after MI, compared to that in sham group. Double immunofluorescence staining was performed to assess the localization of Sirt3 in CD68^+^ macrophages (**Figures 5A, B**). Kyn supplementation reduced the cardiac function in MI models *in vivo*; it increased the infiltration of macrophages in MI region. In mice, the Sirt3 activator viniferin significantly reduced the area of MI and improved the cardiac function following MI. It reduced the infiltration of macrophages in MI site, indicating that the Sirt3 activator can decrease the inflammatory response in the lesion area of MI mice (**Figure 5C**).

### Sirt3 deficiency increased mitochondrial ROS formation and macrophage proinflammatory responses, while overexpression downregulated Kyn-induced alterations in mitochondrial bioenergetics

**Figure 6:**
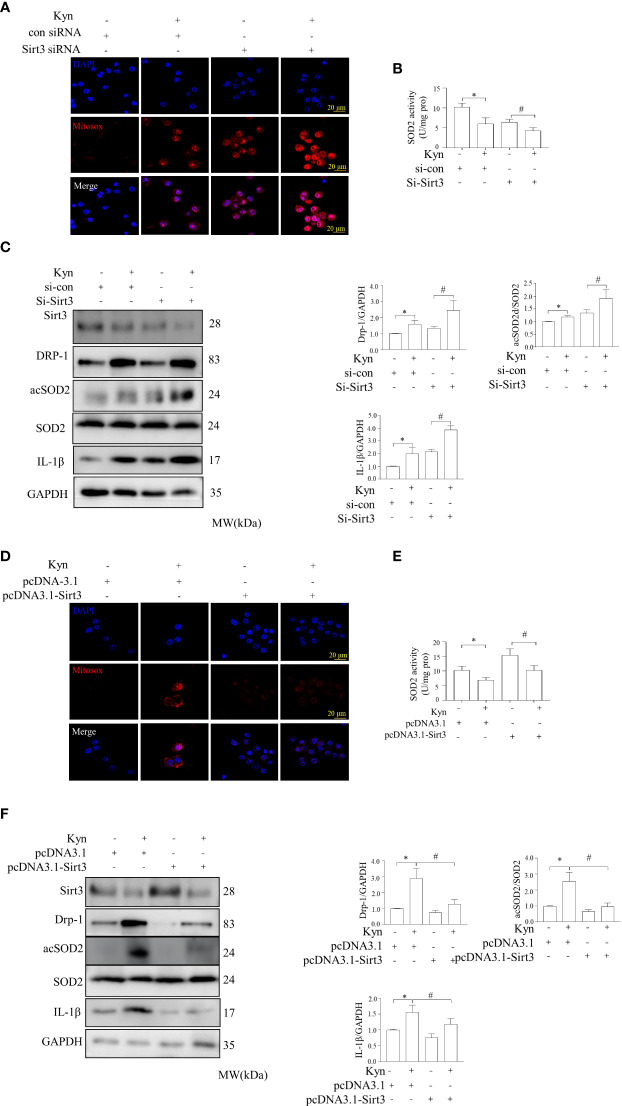
Downregulation and overexpression of Sirt3 altered the levels of inflammatory factors and oxidative stress levels in macrophages induced by Kyn. **(A, B)** Immunofluorescence staining showed that Sirt3 downregulation reduced the levels of mitochondria-derived ROS induced by Kyn. Mitochondria-derived ROS was detected using the MitoSox-probe and imaged using confocal microscopy. SOD activity was detected using the superoxide dismutase test kit. Macrophages transfected with sicon or siSirt3 were treated with Kyn. sicon was the negative control siRNA, while siSirt3 referred was the siRNA against Sirt3, scale bar, 10 μm, *:P < 0.05 vs.si-con; #P < 0.05 vs. Si-Sirt3, n=3. **(C)** Representative blots and quantification of acSOD2, Sirt3, IL-1β, and DRP-1 expression following Kyn stimulation, transfected with negative control, *:P < 0.05 vs.si-con; #P < 0.05 vs. to Si-Sirt3, n=3. **(D, E)** Sirt3 upregulation decreased the levels of mitochondria-derived ROS induced by Kyn, as observed through immunofluorescence. Mitochondria-derived ROS and SOD activity was detected using MitoSox-probe and imaged using confocal microscopy. SOD activity was detected using the superoxide dismutase test kit. The pcDNA3.1 was used as the negative control, and pcDNA3.1-Sirt3 was used for the overexpression of Sirt3. Sirt3 overexpression decreased the levels of mitochondria-derived ROS in macrophages, as observed using the MitoSox-probe and imaged using confocal microscopy, *:P < 0.05 vs. pcDNA-3.1; #P < 0.05 vs pcDNA-3.1 Sirt3, n=3. **(F)** Representative blots and quantification of acSOD2, Sirt3, IL-1β, and DRP-1 expression following the stimulation with Kyn and transfection with pcDNA-3.1 or pcDNA-3.1-Sirt3, *:P < 0.05 vs. pcDNA-3.1; #P < 0.05 vs pcDNA-3.1 Sirt3, n=3.

We determined whether Sirt3 deficiency altered oxidative stress and inflammatory reactions in Raw264.7 macrophage cells. Sirt3 deficiency augmented the oxidative stress and inflammatory reaction stimulated by Kyn in macrophages; there was a marked increase in IL-1β and acSOD2 production, compared to that in control group. Immunofluorescence staining showed that Sirt3 downregulation increased mitochondrial ROS, as observed using specific MitoSox-probes. SOD activity analysis indicated that Sirt3 deficiency increased Kyn-stimulated oxidative stress and inflammatory response in Raw264.7 macrophage cells. Therefore, the deficiency of Sirt3 leaded to enhanced oxidative stress and inflammatory response in macrophage cells, partially by activating the Sirt3-acSOD2/IL-1β signal pathway (**Figures 6A–C**). The overexpression of Sirt3 reduced oxidative stress and inflammatory response in Raw264.7 macrophage cells by downregulating IL-1β and acSOD2. SOD activity analysis indicated that the overexpression of Sirt3 improved Kyn-stimulated oxidative stress and inflammatory response in the Raw264.7 macrophage cells. Mitochondrial derived ROS was downregulated, as observed through MitoSox-probing (**Figure 6D–F**).

### Sirt3 and acSOD2 expression in STEMI patients

**Figure 7:**
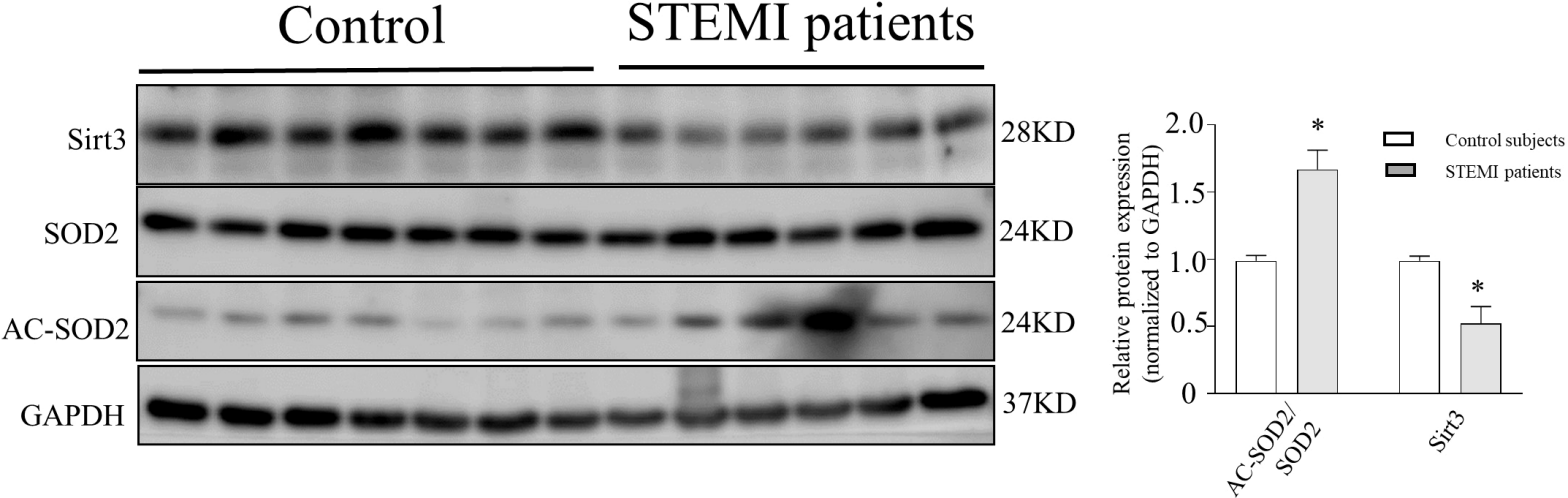
Representative western blots of SOD2 acetylation, SOD2, and Sirt3 expression in PBMCs of STEMI subjects and control subjects. Representative western blots of SOD2 acetylation, SOD2, and Sirt3 expression in STEMI patients compared with that in control subjects. Human peripheral blood mononuclear cells (PBMCs) were obtained from STEMI patients and control subjects. SOD2 acetylation was measured using anti-acetyl-SOD2, *:P < 0.05 vs. control subjects.

ELISA analysis indicated that plasma IDO1 was upregulated in the STEMI patients compared to that in the controls. Western blot analysis of peripheral blood mononuclear cells (PBMCs) from STEMI patients showed that the levels of acSOD2 acetylation increased by 1.68-fold and the activation levels of Sirt3 protein decreased by 1.92-fold compared to that in the control group. Therefore, Sirt3 activity and acSOD2 levels play a pathophysiological role in the occurrence of STEMI (**Figure 7**).

## Discussion

STEMI, the most commonly diagnosed cardiovascular disease, is a leading cause of mortality worldwide. STEMI patients in the highest Kyn quartile had a greater risk of MACCE than these in the lowest quartile. Therefore, Kyn is a risk indicator that could provide prognostic information in patients with STEMI. Kyn is involved in the pathogenicity of STEMI, further corroborating its usefulness in STEMI prognostic analysis. Kyn is a signaling molecule that triggers oxidative stress and inflammatory responses in macrophage cells *via* a Sirt3-acSOD2/IL-1β dependent pathway. Therefore, plasma Kyn could be a potential target for treating STEMI.

However, high-throughput metabolomics may not be ideal for identifying the plasma biomarkers during cardiovascular disease events (8–10). Unbiased studies using untargeted metabolomics combined with functional assessments are important platforms in the field of cardiovascular research. This could enable the identification of meaningful biomarkers for cardiovascular disease (7). STEMI patients have a high mortality in the early stage, particularly during the first year after MI, therefore, selecting the appropriate metabolomic biomarker for identifying patients at high risk and prolonging surveillance is critical. Metabolic data required for the early diagnosis and late prognosis of patients with STEMI are lacking. Therefore, it is difficult to understand the metabolomics signature and integrate functional studies into the pathogenesis of AMI (4, 5). Most of the identified differential metabolites were associated with energy metabolism during STEMI events. This is the first study to apply untargeted metabolomics to delineate the metabolic landscape of patients based on the prognosis of STEMI. Kyn was the most significantly upregulated metabolite in STEMI patients. The highest Kyn quartile of STEMI patients had the greatest risk of MACCE compared to patients in the lowest quartile of 1-year follow-up. Therefore, Kyn may be prognostically valuable for patients with STEMI. The results remained significant after adjustment for conventional risk factors and for contemporary laboratory and echocardiographic factors used for risk stratification, such as high-sensitivity cTnT, NT-proBNP, and LVEF. Zara C et al. (18) also reported that reduced expression of IDO1 and Kyn in monocytes derived dendritic cells (DCs) of NSTEMI patients may exacerbate a chronic inflammatory microenvironment by polarizing the immune response towards effector Th1 lymphocytes. Thus the reduced expression of IDO1 and Kyn in DCs of NSTEMI patients may lead to activate the inflammatory reaction. Therefore, Kyn could be a prognostic marker for STEMI, and reducing Kyn levels could be a promising therapeutic strategy for patients with STEMI. To elucidate the potential functional mechanism by which Kyn exaggerated the pathological process of STEMI, we focused on inflammatory response and oxidative stress. The Kyn pathway contributes to several fundamental biological processes associated with cardiovascular diseases. Melhem et al (19) showed Kyn induces cardiomyocyte apoptosis through the production of reactive oxygen species. This study highlighted a previously unknown causal role of plasma Kyn in inducing inflammation and oxidative stress response in macrophage cells which enhanced the occurrence of STEMI. Therefore, increased Kyn levels contributed to the development of STEMI and Kyn could be a therapeutic target against STEMI. In MI models, plasma Kyn expression were significantly enhanced, and administration of Kyn aggravated the MI phenotype in mice. Therefore, plasma Kyn levels could be used as a biomarker to discriminate STEMI patients with STEMI from control subjects.

The cytological origin of Kyn following the STEMI event is not fully understood. Kyn is upregulated mostly in dendritic cells and macrophage cells in response to proinflammatory stimuli, such as TNF-α,IL-6, and IFN-γ (16, 20, 21). Kyn metabolites increased inflammation, oxidative stress, and apoptosis in smooth muscle and endothelial cells. Melhem et al. found that Kyn, produced by endothelial cells, induced the apoptosis of cardiomyocytes through reactive oxygen species production in MI models. However, complex interactions among different cells, such as cardiomyocytes, inflammatory cells, and vascular cells, regulate tissue remodeling following MI. The inflammatory profile of macrophage cells are highly dependent on their metabolic state particularly in case of proinflammatory macrophage cells (11, 16). Therefore, inflammatory cells, especially macrophages recruited in the thrombotic tissue and necrotic myocardial tissue following the release of Kyn, would aggravate the progression of MI. Macrophage cells in infarcted areas may produce and release Kyn into the plasma, leading to an inflammatory cascade effect in the infarcted regions. Therefore, we focused on macrophages to determine the role of Kyn in promoting the development of STEMI and the potential regulatory mechanism of Kyn. We hypothesized that disrupting the key metabolic pathway of Kyn production in macrophages would inhibit the development of STEMI. Therefore, IDO1 could be a potential therapeutic target for STEMI, and reducing the IDO1 expression in macrophage cells would reduce the plasma levels of Kyn in STEMI patients.

Kyn acts as a ligand that binds to various molecules and induces the corresponding biological effects. Through prediction analysis, we find that Kyn acts as a ligand binding to Sirt3, a NAD^+^-dependent mitochondrial deacetylase that acts as a metabolic sensor to modulate mitochondrial function to match nutrient supply (22, 23). Sirt3 inactivation can lead to hyperacetylation at the SOD2 K89 site and promote vascular oxidative stress and inflammatory response (24). Kyn reduced Sirt3 expression *in vitro* at the transcriptional level in macrophages, in addition, it inhibited the inflammatory response and oxidative stress in macrophages. Sirt3 levels were decreased, while acSOD2 expression was increased in the PBMCs of STEMI patients, indicating that Kyn functions by upregulating the Sirt3-acSOD2 signaling pathway in the inflammatory and oxidative stress responses of macrophage cells.

Aryl hydrocarbon receptor (AhR) (25), a receptor of Kyn, is a ligand-activated transcription factor involved in a series of crucial pathological processes, especially immune and inflammatory responses (26, 27). Changes in the expression or function of AhR during the pathophysiology of STEMI remains unknown. Future studies should focus on verifying whether the Kyn–AhR axis is involved of STEMI progression. Neutralizing antibodies against Kyn production could be a possible strategy against the progression of STEMI.

There are several potential limitations in this study. (1) First ^1^H-NMR revealed 15 differentially expressed metabolites with structural diversity related to STEMI patients. The role of other metabolites such as alanine, glutamate, histidine, leucine, isoleucine, and valine, which had significant P values, was not evaluated. Future studies would focus on elucidating the correlation between other meaningful metabolites and STEMI. (2) Plasma Kyn concentrations were measured using the UPLC/Q-TOF; therefore, measurement of Kyn levels using a routine laboratory analyzer should be standardized for clinical use. (3) Due to the limited sample size and occurrence of MACCE in the STEMI cohort, this result cannot be generalized to the overall population and should be validated in a larger cohort. In addition, we followed-up the STMEI patients for only 1 year; therefore, we could not examine the relationship between the dynamic changes in Kyn levels and long-term outcomes. (4) Finally, although we adjusted our analysis for various potential confounders, we cannot exclude the influence of unknown or unmeasured confounders.

In conclusion, plasma level of Kyn was associated with increased risk of MACCE events in STEMI patients. Kyn could be useful for determining the outcomes in STEMI patients. Kyn could interrupt the oxidative stress and inflammatory reaction in macrophage cells through the Sirt3-acSOD2/IL-1β pathway. Therefore, Kyn-related pathways could be a new target for STEMI treatment. Further investigations with larger sample sizes and/or in other cohorts are encouraged to confirm the value of Kyn as a novel cardiac biomarker and to design future clinical applications using Kyn as a predictive indicator of STEMI events.

## Data availability statement

The original contributions presented in the study are included in the article/**Supplementary Material**. Further inquiries can be directed to the corresponding authors.

## Ethics statement

The studies involving human participants were reviewed and approved by The General Hospital of Northern Theater Command. The patients/participants provided their written informed consent to participate in this study. The animal study was reviewed and approved by The General Hospital of Northern Theater Command.

## Author contributions

YH was responsible for the study and design the research. XZ wrote the manuscript. YC and XS took the targeted analysis for tryptophan and its metabolites by LC-MS/MS. KN and MQ provided the statistical analysis. XT, DL, CY did the vivo and in vitro experiments. QJ and HL collected clinical cases. TW did the molecular docking study. All authors critically revised the paper and approved the final manuscript.

## Funding

The Major special projects of social development in Liaoning Province (Grant No. 2020JH1/10300002), 2021 Provincial Key research and development Plan Joint Plan (2021JH2/10300128) and the National Key Research and Development Program of China (2016YFC0900904).

## Conflict of interest

The authors declare that the research was conducted in the absence of any commercial or financial relationships that could be construed as a potential conflict of interest.

## Publisher’s note

All claims expressed in this article are solely those of the authors and do not necessarily represent those of their affiliated organizations, or those of the publisher, the editors and the reviewers. Any product that may be evaluated in this article, or claim that may be made by its manufacturer, is not guaranteed or endorsed by the publisher.

## Abbreviations

STEMI, ST-acute myocardial infarction; NSTEMI, non-ST-segment elevated myocardial infarction; AMI, acute myocardial infarction; MI, myocardial infarction; CAD, coronary artery disease; SAP, stable angina pectoris; AUC, area under the curve; CK-MB, creatine kinase isoenzyme; CRP, C-reactive protein; MACCE, major adverse cardiac and cerebral events ; CV, cardiovascular; LVEF, left ventricular ejection fraction ; MACCE, major adverse cardiovascular events ; proBNP, pro–B-type natriuretic peptide; hscTnT, high-sensitivity cardiac troponin T ; 1H NMR, 1H Nuclear magnetic resonance; UPLC/Q-TOF, Ultraperformance liquid chromatography/electrospray ionization quadruple time-of-flight mass spectrometry; MACCE, Major Adverse Cardiovascular Events; Trp, Tryptophan; Kyn, Kynurenine; IDO1, indoleamine-pyrrole 2,3-dioxygenase, AhR, Aryl hydrocarbon receptor.
