# Corrigendum: Untargeted metabolomics identified kynurenine as a predictive prognostic biomarker in acute myocardial infarction

**DOI:** 10.3389/fimmu.2023.1163419

**Published:** 2023-03-08

**Authors:** Xiaolin Zhang, Yi Cai, Xu Su, Quanmin Jing, Haiwei Liu, Kun Na, Miaohan Qiu, Xiaoxiang Tian, Dan Liu, Tianxiao Wu, Chenghui Yan, Yaling Han

**Affiliations:** ^1^ Second Affiliated Hospital of Dalian Medical University, Dalian, China; ^2^ Cardiovascular Research Institute and Department of Cardiology, The General Hospital of Northern Theater Command, Shenyang, China; ^3^ Key Laboratory of Structure-Based Drug Design and Discovery, Ministry of Education, School of Pharmaceutical Engineering, Shenyang Pharmaceutical University, Shenyang, China

**Keywords:** ST-acute myocardial infarction, Kynurenine, metabolites, prognosis, biomarker, indoleamine-pyrrole 2,3-dioxygenase

In the published article, there was an error in affiliation 1. Instead of “Department of Cardiology, Dalian Medical College, Dalian, China”, it should be “Second Affiliated Hospital of Dalian Medical University, Dalian, China”.

The authors apologize for this error and state that this does not change the scientific conclusions of the article in any way. The original article has been updated.

