# Supplementary material for: Untargeted metabolomics identified kynurenine as a predictive prognostic biomarker in acute myocardial infarction
Source: Front Immunol. 2022 Nov 2;13:950441. doi: 10.3389/fimmu.2022.950441 (PMC9667794; doi:10.3389/fimmu.2022.950441)
Supplement: Supplementary file 1 [file DataSheet_1.pdf]

Untargeted metabolomics identified Kynurenine as a predictive prognostic biomarker in acute myocardial infarction

Short title: Xiaolin Zhang, et al. Kynurenine as a predictive prognostic biomarker in acute myocardial infarction

Xiaolin Zhang[1,2,*], Yi Cai[2,*], Xu Su[2,*], Quanmin Jing[2], Haiwei Liu[2], Kun Na[2], Miaohan Qiu[2], Xiaoxiang Tian[2], Dan Liu[2], Tianxiao Wu[3], Chenghui Yan[2,#], Yaling Han [1,2,#]

1: Department of Cardiology, Dalian Medical University, Dalian Liaoning Province, China
2: Cardiovascular Research Institute and Department of Cardiology, The General Hospital of Northern Theater Command, 83 Wen Hua Road, Shenyang Liaoning Province, China
3: Shenyang Pharmaceutical University, Key Laboratory of Structure-Based Drug Design and Discovery

*: These authors contributed equally as first author
#: corresponding author

Running Title: Kynurenine as a predictive prognostic biomarker in acute myocardial infarction

Supplemental Methods 3-6
Supplemental Results 7-8
Supplemental Tables 9-20
Supplemental Figure Legends 21
Supplemental References 22-23

ONLINE SUPPLEMENTAL MATERIAL

MATERIALS and METHODS

Study design and participants

In order to clarify the characteristics of plasma metabolism in STEMI patients, 50 consecutive STEMI patients presenting with acute chest pain to the emergency department and 50 control subjects were recruited in the General Hospital of Northern Theater Command from April 2014 to June 2014 for the ^{1}H-NMR analysis **(Supplemental Figure 1)**. The baseline characteristics and clinical parameters were shown in **Supplemental Table1.** STEMI patients[1,2] were diagnosed according to the American College of Cardiology/American Heart Association (ACC/AHA) Guidelines.

In order to further verify the results of ^{1}NMR analysis, a total of 520 participates were enrolled in our study. At last a total 116 control subjects, 132 stable angina pectoris (SAP) patients,124 non-ST-segment elevated myocardial infarction (NSTEMI) patients and 148 STEMI patients were recruited in the General Hospital of Northern Theater Command from April 2014 to October 2014 to evaluate the Trp and its metabolites concentration in the variation stage **(Supplemental Figure 1)**. The baseline characteristics and clinical parameters were shown in **Table 1**. The STEMI patients with a higher prevalence of hypertension were more likely to be smokers and had the highest white blood cell counts. The inclusion criteria of STEMI patients and control subjects were the same as above. NSTEMI and SAP was were diagnosed according to the American College of Cardiology/American Heart Association (ACC/AHA) Guidelines[3,4.]

Follow-up and End Points

As a whole, we further analyzed the prognostic value of Kyn in the 977 STEMI recruited in the General Hospital of Northern Theater Command from January 2015 to April 2017 **(Supplemental Figure 1, Table 2)**. All 977 STEMI patients were followed up by telephone every 6 months and via outpatient clinic visits for 1 year.

Metabolomics analysis based on ^{1}H NMR

The ^{1}H NMR analysis was done by Shanghai Metabolome Institute-Wuhan (SMI) (ShangHai, China). In total 50 STEMI patients and 50 control subjects were selected respectively to analyse the characteristics of plasma metabolism. PCA, PLS-DA and OPLS-DA analysis were acquired by the Simca-P+13.0 software[5].

Targeted analysis for tryptophan and its metabolites by LC-MS/MS

Liquid chromatographic separation for processed plasma was achieved on a UPLC HSS C_{18} column (100mm×2.1mm,1.7μm) using a UPLC/Q-TOF (Waters Corp., Milford, MA, USA)[6,7].The analyte specific settings were described in **Supplemental Table 2-9**.

Biomarker Measurement

Blood samples were collected in plasma separator with the EDTA-anticoagulation[8]. Enzyme-linked immunosorbent assay (ELISA) was using to detect the IDO1 level of STEMI and control subjects (Novus NBP2-62765, American).

Human Thrombi Tissues in STEMI patients

Human coronary artery thrombi were obtained using a thrombectomy catheter (Export catheter, Medtronic, Minneapolis, MN, USA) in STEMI patients at the time of percutaneous coronary intervention.The coronary thrombus tissues from STEMI patients were obtained by aspiration catheter.

Isolation of human peripheral blood monocytes

We withdrew samples of human peripheral blood monocytes (PBMCs) from STEMI patients and control subjects, and then isolated PBMCs and resolved by a ficoll-density-gradient separation. Furthermore by western blot analysis the expression of Sirt3, SOD2 and ac-SOD2 were detected in PBMCs [9].

Materials

We obtained all chemicals such as Tryptophan (574597), 3-Hydroxy-DL-kynurenine (H1771), anthranilic acid (36941), Kynurenic acid (67667), xanthurenic acid (D120804), and quinolinic acid (BBO000002) from Sigma Aldrich (St Louis, MO, USA). Antibodies for the detection of IDO1 (ab131086), SOD2(ERP2560Y), IL-1β(ERP23851-127), DRP-1(ERP19274), GAPDH (AB8245), ac-SOD2(AB137037) and CD68 (ERP23917-164) were from Abcam (American). We purchased antibodies for Sirt3(5490) from Cell Signaling Technology (Danvers, MA, USA). All primary antibodies were used in a 1:1,000 dilution for Western blot and a 1:100 dilution for immunocytochemistry and immunohistochemistry. IFN-γ recombinant human protein (PHC4031) was obtained from Gibco. Human IDO1 (NBP-62765) ELISA kits were from NOVUS Biosciences. For inhibition of Sirt3, small interfering RNA (siRNA) and negative control sequences were obtained from RiboBio (Guangzhou, China). The transfection reagents for siRNA (Lipofectamine RNAiMax, 13778150) were from Invitrogen. For the overexpression Sirt3, pCDNA3.1-Sirt3 plasmid and its control were constructed and purchased from GENEWIZ technology corporation (Suzhou, China). Transient transfection was performed by using lipofectamine™ RNA iMAX and lipofectamine 2000 transfection reagent (Thermo, USA) for 24 h, according to the manufacturer's instructions.

Animal studies

Treatment and groups

To examine the IDO1 and Sirt3 function, C57 mice were divided into sham group (WT+Sham) and myocardial infarction group (WT+MI) [10]. All animal experiments were approved by the Committee on Animal Resources of the General Hospital of Northern Theater Command.

Echocardiographic and hemodynamic analyses of cardiac function

Echocardiographic and hemodynamic analyses of cardiac function between the sham and MI group were analysed by the transthoracic echocardiography Vevo 2000. In order to keep the 550–650 bpm heart rate, and the mice were slightly anesthetized.

Cell cultures and experiments in vitro protocol

Cytological expression profiling showed that IDO1 was highly expressed in macrophage cells. Furthermore we obtained the macrophage cells (Raw 264.7) from the American Type Culture

Collection (ATCC, Manassas, VA, USA) and cultured them in RPMI 1640 medium. IDO1 expression in macrophage cells stimulated with recombinant IFN γ (20 ng/ml) for 24 h was significantly upregulated. We grew the macrophage cells to sub-confluence and then stimulated them with the Kyn to explore concentration-response and time-response curve according to 0, 1,5,10,15 μmol and 0, 2, 4, 6, 8, 12, 24 h differently. Thus we found that 15 μmol Kyn simulated macrophage cells for 24 h can upregulated the acSOD2 expression and downregulated the Sirt3 expression obviously.

In Vivo siSIRT3 and pcDNA3.1-Sirt3 Delivery

The siRNA or siSirt3 (RiboBio) were transfected using the lipofectamine™ RNA iMAX transfection reagent. Furthermore the CDS region of Sirt3 (mouse) gene was built and transferred into the pcDNA3.1 vector synthesized by GENEWIZ (Suzhou, China). We used the DNA endotoxin-free plasmid purification kits (Promega, Madison, WI) to extract the plasmids and delivered the plasmids into the macrophage cells with lipofectamine 2000 transfection reagent.

Immunohistochemical analyses and Immunofluorescence Staining

Immunohistochemical analyses were performed using paraffin-embedded tissue sections and the sections were incubated with primary anti-IDO1 and anti-CD68 antibodies at 4°C overnight. Following the incubation, a Vectastain Elite ABC kit (ZSGB-BIO 9720, China) was used according to the manufacturer's instructions. The images were observed and photographed under a microscope (ZEISS image A2, Germany) or a confocal laser scanning microscope (ZEISS 800, Germany).

MitoSOX Red Stain

To examine mitochondrial reactive oxygen species (ROS) levels, macrophage cells were loaded with 5 μM MitoSOX Red (30 min, 37 °C) (M36008, Invitrogen, USA) which was a mitochondrial superoxide indicator [11]. The nucleus was stained with Hoechst 33342 for 5 min at 37 °C (H3570, Thermo Fishery Company, USA). The cells were live-imaged immediately and photographed with the confocal laser scanning microscope (ZEISS 800, Germany).

Detection of superoxide dismutase (SOD)

To examine mitochondrial reactive oxygen species (ROS) levels, cells were loaded with 5 μM MitoSOX Red for 10 min at 37 °C, which was a mitochondrial superoxide indicator using commercial assay Kits (Solarbio Chemical Company, China).

Molecular docking study

The Sirt3 crystal structure (PDB code: 4BN4) which was downloaded from protein data bank (https://www.rcsb.org/) was processed with the Protein Preparation Wizard in the Schrçdinger suite. The protein structure was adjusted and modified followed by adding hydrogen atoms, deleting solvent water molecules, and defining right bonds orders using Prime. The protonation and tautomeric states of Asp, Lys, and His were assigned at pH 7.4 state. Afterward, all hydrogen atoms of Sirt3 complexes were optimized with OPLS_2005 force field, which minimized and converged heavy atoms to a RMSD of 0.3. The selected inhibitors were prepared by using LigPrep from the

Schrçdinger suite with the OPLS_2005 force field. The structure of inhibitors was also adjusted and modified, followed by adding all hydrogen atoms, checking the bond order and atom types.

Receptor grids were generated before docking with allosteric site determined by the literatures. The prepared protein–ligand complex was imported into Glide 9.7, which defined it as the receptor structure with size box (20Å× 20Å ×20Å). Based on the OPLS_2005 force field, the grid of TRKA crystal structure was generated. The extra precision (XP) mode was set for docking studies.

Ethics, consent and permissions

All procedures were designed according to the declaration of Helsinki's. The study protocol was ethically reviewed and approved by the Ethics Review Committee of the General Hospital of Northern Theater Command. Signed informed consent was obtained from all subjects prior to their inclusion in the study.

Statistical analysis

Categorical variables were reported as counts and percentages, and between-group differences were assessed with chi-square or Fisher's exact test. Continuous variables were presented as the mean ± SD and were compared with one-way analysis of variance. We examined the recruited patients characteristics, treatments, tests, procedures, and crude rate of outcomes across quartiles of concentration Kyn using the Cochran-Armitage trend test for the trend of binary variables, and the Mann-Kendall trend test for trends of continuous variables. Survival curves for time-to-event variables were compared by the log-rank test. Receiver operating characteristic curve for concentration Kyn was constructed to assess the predictive accuracy for 1-year MACCE. The non-linear association was further evaluated by using restricted cubic splines between concentration Kyn and 1-year MACCE. The logistic regression was used to investigate the independent predictors for MACCE at 1 year. Statistical analysis was performed using SPSS 23.0 (IBM SPSS Inc., Chicago, IL, USA) and R-3.6.3 (R Core Team, Vienna, Austria). A two-tailed $P<0.05$ was considered statistically significant.

EXTENDED RESULTS

Results

Baseline characteristics of the study population

In this study, 50 control subjects and 50 STEMI patients with time of onset less than 3 h were collected for the analysis of tryptophan and its metabolites. **Supplemental table 1** illustrated the baseline characteristics of 50 STEMI patients and 50 participates. Biochemical results showed that triglyceride, HDL-C level and LDL-C had no statistical significance between the control subjects and STEMI patients ($P>0.05$). There were significant differences in hs-CRP value, hscTnT and CK-MB value between the control subjects and STEMI patients ($P<0.05$), as shown in **Supplemental Table1**.

The typical ^{1}H-NMR spectra of plasma showed that the structure analysis of metabolites in the plasma of 50 control subjects and 50 STEMI patients combined with the information provided by ^{1}H-NMR spectra and a series of 2D NMR spectra. The metabolites spectra were showed in the Supplemental Figure 2. 35 main metabolites among them 31 metabolites were known and 4 were unknown were identified in the selected population (**Supplemental Table 2**). Correlation coefficients for the most predominant altered metabolites and metabolic pathways were shown significantly differences between control subjects and STEMI participants (**Supplemental Table 3-4**). Furthermore targeted analysis for tryptophan and its metabolites by LC-MS/MS was used for further analysis in the control subjects and STEMI patients. The seven gradients of the standard curve of the Trp, Trp-d3, Kyn, KA, 3-HK, 3-HAA, QA and XA were prepared by diluting them step by step with ultrapure water (**Supplemental Table 5,6**). Trp and Kyn, KA, 3-HK, 3-HAA, XA and QA concentrations were 500-40000, 25-2000, 6.25-500, 6.25-500, 6.25-500, 6.25-500, 25-2000 ng/mL differentially. The results showed that the method had the high sensitivity and meet the requirements of subsequent samples (**Supplemental Table 7**). The targeted LC-MS/MS method was validated shown in **Supplemental Table 8**. The stability of the plasma sample is shown in **Supplemental Table 9.** Supplemental Figure 3 showed the representative chromatograms of detection of amino acids 3-HAA, XA, Trp, KA, TRP, Kyn, QA and 3-HK on positive ion mode.

Tissue and cytological expression of IDO1

IDO1 expression profiles in the different tissues of mice indicated IDO1 expressed in the heart tissue. (**Supplemental Figure 4**).

IDO1 expression was high in coronary artery plaque

To evaluate the changes in IDO1 expression in human atherosclerotic coronary arteries, we compared IDO1 expression in coronary arteries plaque with normal coronary arteries. The expression of IDO1 in the macrophage cells of coronary artery was identified through immunostaining of serial sections. In normal coronary vessels, there were no CD68 positive macrophage cells and the IDO1 expression was low. However, in the atherosclerotic coronary artery plaque, CD68+ macrophages were present and the IDO1 expression was high, compared to the control (**Supplemental Figure 5A)**. Immunofluorescence staining indicated that the $CD68^+$

macrophages were co-localized with IDO1 in the coronary artery plaque (**Supplemental Figure 5B)**.

Molecular Docking

To investigate the interaction mode of Kyn and Sirt3, a docking study was performed. The best predicted binding mode was shown in Figure 4E, and the binding energy was -5.794 kcal/mol. The carboxyl group at the Kyn terminus forms salt-bonding interactions with Arg-158 and hydrogen-bonding interactions with Phe-157 and Arg-158. The benzoyl group and the protonated amino group were form a cross-linked hydrogen bond network with Gly-319, Thr-320 and Ser-321. Further stabilization was achieved through the π-π stacking (edge to face) interaction of the benzene ring with the Phe-157.

LEGEND TO ONLINE SUPPLEMENTAL TABLES AND FIGURES

Supplemental Table 1. Baseline characteristics of the cohort in discovery population

Variable	Control subjects n=50	STEMI patients n=50	P-value
Age, y	58.4±12.2	60.5±10.7	0.302
Males, n (%)	32(64.0)	36(72.0)	0.095
Blood Pressure, mmHg	127.2±15.3	132.2±11.7	0.157
Heart Rate, bmp	77.4±16.3	86.2±16.3	0.487
Smoking, n (%)	29(58.0)	33(66)	0.119
Hypertension, n (%)	28(56)	31(62)	0.251
Diabetes, n (%)	10(20.0)	13(26.0)	0.159
LVEF, %	62.2±8.4	59.2±11.4	0.053
TG, mmol/L	1.9±1.3	2.1±1.1	0.172
TC, mmol/L	4.8±1.3	4.9±1.5	0.366
HDL-C, mmol/L	1.0±0.2	0.9±0.2	0.096
LDL-C, mmol/L	2.9±1.2	3.0±0.9	0.104
GLU, mmol/L	6.3±2.5	7.7±4.3	0.159
WBC, 109/L*	6.9±1.7	11.5±3.4	<0.001
Hs-CRP, mg/L*	1.0±0.7	3.1±6.0	0.001
hscTnT, mg/L*	0.01±0.02	0.95±2.00	<0.001
CK-MB, U/L*	10.3±5.0	91.1±100.1	<0.001

Data are expressed as mean ± SD, or n (%), LVEF, left ventricular ejection fraction; TG, triglycerides; TC, total cholesterol; LDL-C，low density lipoprotein cholesterol; HDL-C，high density lipoprotein cholesterol; GLU, blood glucose; WBC, white blood cell; Hs-CRP, high-sensitivity C-reactive protein; hscTnT, high-sensitivity troponin T; CK-MB, creatine kinase MB isoenzyme.

Supplemental Table 2. Summary of ^{1}H-NMR analysis identified metabolites in plasma samples from control subjects and STEMI patients

Number	Metabolites	Moieties	^{1}H (ppm) and multiplicity [a]	^{13}C
1	Leucine (Leu)	$\underline{C}H_3$	0.94(d)	24.1
		'$\underline{C}H_3$	0.96(d)	25.0
		$\underline{C}H_2$	1.69(m)	42.7
		$\underline{C}H_2$	1.72(m)	27.0
		$\underline{C}H$	3.76(t)	56.5
		$\underline{C}$OOH	-	178.6
2	Isoleucine (Ileu)	$\underline{C}H_3$	0.93(t)	14.1
		C$\underline{C}H_3$	1.00(d)	17.6
		$\underline{C}H_2$	1.26(m)	27.1
		$\underline{C}H_2$'	1.46(m)	27.1
		$\underline{C}H$	1.99(m)	38.8
		$\underline{C}H$	3.68(d)	63.1
		$\underline{C}$OOH	-	177.1
3	Valine (Val)	$\underline{C}H_3$	0.99(d)	19.5
		'$\underline{C}H_3$	1.04(d)	20.9
		$\underline{C}H_2$	2.27(m)	32.1
		$\underline{C}H$	3.61(d)	63.3
		$\underline{C}$OOH	-	177.3
4	3-Hydroxybutyric acid (3-HB)	$\underline{C}H_3$	1.20(d)	24.5
		$\underline{C}H_2$	2.31(dd)	49.8
		$\underline{C}H_2$'	2.41(dd)	49.8
		$\underline{C}H$	4.16(m)	68.4
		$\underline{C}$OOH	-	183.3
5	Lactate (Lac)	$\underline{C}H_3$	1.33(d)	22.9
		$\underline{C}H$	4.12(q)	71.6
		$\underline{C}$OOH	-	185.4
6	Alanine (Ala)	$\underline{C}H_3$	1.48(d)	19.2
		$\underline{C}H$	3.79(q)	53.4
		$\underline{C}$OOH	-	179.0
7	Acetate	$\underline{C}H_3$	1.92(s)	26.5
		$\underline{C}$OOH	-	184.1

8	Glutamate (Glu)	CH_2	2.06(m)	36.5
		CH_2	2.13(m)	30.1
		CH_2’	2.34(m)	30.1
		CH	3.76(t)	57.6
		COOH	-	nd
9	Citrate	CH_2	2.53(d)	48.6
		CH_2’	2.66(d)	48.6
10	Creatinine	CH_3	3.05(s)	33.0
		CH_2	4.06(s)	59.2
11	Choline	CH_3	3.21(s)	56.5
		CH_2-NH	3.52(m)	70.5
		CH_2-OH	4.07(m)	57.6
12	β-glucose (β-Glc)	2-CH	3.25(m)	76.8
		4-CH	3.41(m)	72.6
		3,5-CH	3.47(m)	78.3
		6-CH	3.75(dd)	63.5
		6-CH’	3.90(dd)	63.5
		1-CH	4.66(d)	95.0
13	α-glucose (α-Glc)	4-CH	3.40(m)	71.8
		2-CH	3.54(dd)	74.3
		3-CH	3.73(m)	75.0
		5-CH	3.83(m)	75.2
		6-CH_2	3.84(m)	64.0
		1-CH	5.24(d)	95.0
14	Tyrosine (Tyr)	CH_2	3.05(m)	38.7
		CH_2’	3.18(m)	38.7
		CH	3.95(m)	59.1
		3,5-CH	6.91(d)	118.6
		2,6-CH	7.20(d)	133.4
		COOH	-	nd
15	Histidine (His)	CH_2	3.07(dd)	nd
		CH_2’	3.13(dd)	nd
		CH	3.96(dd)	nd
		4-CH	7.08(s)	120.0
		2-CH	7.83(s)	139.1
		COOH	-	nd
16	Phenylalanine (Phe)	CH_2	3.12(dd)	nd
		CH_2’	3.28(dd)	nd

		$\underline{C}H$	3.99(dd)	nd
		4-$\underline{C}H$	7.33(m)	132.3
		3,5-$\underline{C}H$	7.38(m)	130.8
		2,6-$\underline{C}H$	7.43(m)	132.0
		$\underline{C}OOH$	-	nd
17	Formate (Form)	$H\underline{C}OOH$	8.45(s)	nd
18	Threonine (Thr)	$\underline{C}H_3$	1.34(d)	22.6
		$\underline{C}H$	3.59(d)	63.2
		$\underline{C}H$	4.25(m)	69.0
		$\underline{C}OOH$	-	nd
19	Tryptophan (Trp)	3$\underline{C}H$	7.33(s)	nd
		5$\underline{C}H$	7.20(t)	nd
		6$\underline{C}H$	7.28(t)	nd
		7$\underline{C}H$	7.55(d)	nd
		4$\underline{C}H$	7.74(d)	121.4
		$\underline{C}OOH$	-	nd
20	Glycine (Gly)	$\underline{C}H_2$	3.56(s)	44.7
		$\underline{C}OOH$	-	175.2
21	Lysine (Lys)	$\underline{C}H_3$	1.47(m)	24.0
		$\underline{C}H_3$	1.71(m)	29.2
		$\underline{C}H$	1.90(m)	32.8
		$\underline{C}H_2$	3.02(t)	41.8
		$\underline{C}H$	3.76(t)	57.2
		$\underline{C}OOH$	-	nd
22	Hypoxanthine	2-$\underline{C}H$	8.20(s)	148.6
		8-$\underline{C}H$	8.21(s)	144.8
23	Phosphocholine (PC)	$N(\underline{C}H_3)_3$	3.22(s)	56.8
		N-$\underline{C}H_2$	3.58(m)	nd
		O-$\underline{C}H_2$	4.24(m)	72.3
24	Pyroglutamate (PGlu)	4-$\underline{C}H_2$	2.03(m)	28.2
		4-$\underline{C}H_2$'	2.41(m)	32.4
		3-$\underline{C}H$	2.50(m)	28.4
		5-$\underline{C}H$	4.18(dd)	61.3
		$\underline{C}OOH$	-	183.3

		2’ C*H*	6.10(d)	91.1
25	Inosine	8-C*H*	8.25(s)	149.4
		2-C*H*	8.35(s)	143.2
26	Methanol	CH_3	3.36(s)	51.8
		Ca-EDTA^{2-}	2.56(s)	57.9
		Ca-EDTA^{2-}	3.13(q)	63.9
27	Edetic Acid (EDTA)	Mg-EDTA^{2-}	2.70(s)	58.1
		Mg-EDTA^{2-}	3.24(m)	77.2
		Free-EDTA	3.22(s)	53.9
		Free-EDTA	3.63(s)	60.2
		4,6-C*H*	6.97(m)	nd
28	Salicylate	5-C*H*	7.47(m)	nd
		3-C*H*	7.83(dd)	nd
		COOH	-	nd
		1,4,6-C*H*	3.65(m)	nd
		6-C*H*	3.73(dd)	nd
29	Glucitol	3-C*H*	3.76(m)	nd
		1-C*H*	3.83(d)	nd
		2,5-C*H*	3.85(m)	nd
		CH_3	0.90(t)	11.5
		CH_2	1.64(m)	29.8
30	2-Hydroxybutyric acid (2-HB)	CH_2’	1.73(m)	29.8
		C*H*	4.01(dd)	76.3
		COOH	-	nd
			1.17(b)	30.9
			1.28(b)	nd
31	Lipids		1.77(b)	36.8
			2.05(b)	20.3
			2.35(b)	34.2
U1	Unknown1		3.63(s)	62.8
U2	Unknown2		3.93(s)	56.0
			3.68(b)	66.5
U3	Unknown3		3.79(b)	66.5
			4.05(b)	72.7
U4	Unknown3		1.94(m)	25.0

[a]s, singlet; d, doublet; t, triplet; q, quartet; m, multiplet; dd, doublet of doublet; b, broad peak; nd, not determined

Supplemental Table 3. Correlation coefficients was obviously different between control group and STEMI group

Number	Metabolite	chemical shift (ppm)	correlation coefficient (r)
8	Glu	2.13	+0.83
19	Trp	7.74	+0.78
15	His	7.08	+0.77
29	Glucitol	3.85	+0.69
17	Form	8.45	+0.66
2	Ileu	1.00	+0.63
14	Tyr	6.91	+0.63
1	Leu	0.96	+0.60
3	Val	1.04	+0.56
20	Gly	3.56	+0.53
6	Ala	1.48	+0.42
24	PGlu	2.41	-0.61
4	3-HB	1.2	-0.42
13	a-Glc	5.24	-0.35
28	Salicylate	6.97	-0.33

Glu, glutamate; Trp, tryptophan; His, histidine; Form, formate; Tyr, tyrosine; Leu, leucine; Val, valine; Gly, glycine; Ala, alanine; PGlu, pyroglutamate; 3-HB, 3-Hydroxybutyric acid; a-Glc, α-glucose. The coefficients were from OPLS-DA results; positive and negative signs indicated positive and negative correlations, respectively.

Supplemental Table 4. The most predominant altered metabolic pathways between control subjects and STEMI patients.

Pathway Name	-log(p)	Holm p	FDR	Impact
Alanine, aspartate and glutamate metabolism	5.963	0.198	0.051	0.177
Phenylalanine metabolism	4.721	0.6594	0.102	0.119
D-Glutamine and D-glutamate metabolism	3.323	1.000	0.320	0.112
Tryptophan metabolism	1.450	1.000	0.816	0.109

Supplemental Table 5. Multiple reaction monitoring ion parameters mass spectrometry conditions

Compound	Ion mode	Parent mass(*m/z*)	Daughter mass(*m/z*)	Cone (V)	Collision energy(V)	RT(min)
TRP	+	205.10	188.08	40	14	0.77
KYN	+	209.10	94.06	40	25	0.65
KA	+	190.05	144.05	40	18	0.77
HK	+	225.09	208.07	40	12	0.46
XA	+	206.09	160.05	40	22	0.74
HAA	+	154.10	108.04	40	20	0.74
QA	+	168.03	150.02	40	14	0.75
TRP-d3	+	208.12	191.05	40	14	0.76

Supplemental Table 6. The series of concentration of Trp, Kyn, KA, 3-HK, XA, 3-HAA and QA standards (ng·mL^{-1})

Compound	No.1	No.2	No.3	No.4	No.5	No.6	No.7
TRP	500	1000	2000	5000	10000	20000	4000<br>0
KYN	25	50	100	250	500	1000	2000
KA	6.25	12.5	25	62.5	125	250	500
3-HK	6.25	12.5	25	62.5	125	250	500
XA	6.25	12.5	25	62.5	125	250	500
3-HAA	6.25	12.5	25	62.5	125	250	500
QA	25	50	100	250	500	1000	2000

Supplemental Table 7. Summary of regression equations, linear ranges and LLOQs of the Trp, Kyn, KA, 3-HK, XA, 3-HAA and QA analytes

Analyte	Regression equations	r	Linear range (ng/mL)	LLOQ	
				RSD (%)	RE (%)
TRP	$y = 0.6465x + 6.5686$	0.9991	500-40000	11.5	-6.1
KYN	$y =4.291\times10^{-2} x - 6.017\times10^{-1}$	0.9968	25-2000	6.9	-5.8
KA	$y = 4.740 x +33.06$	0.9970	6.25-500	5.4	2.3
3-HK	$y = 0.2731x -1.286$	0.9917	6.25-500	8.7	4.1
XA	$y = 2.1653x- 0.9306$	0.9986	6.25-500	12.5	8.2
3-HAA	$y = 0.3969 x+1.1936$	0.9957	6.25-500	12.6	-9.2
QA	$y = 2.790\times10^{-2} x + 0.5746$	0.9951	25-2000	10.1	-7.9

Supplemental Table 8. Summary of accuracy, precision, recovery and matrix effect of the Trp and Kyn, KA, 3-HK, XA, 3-HAA and QA analytes

Analytes	Concentration (ng/mL)	Intra-day RSD (%)	Inter-day RSD (%)	Accuracy RE (%)	Recovery (%, mean ± SD)	Matrix effect (%, mean ± SD)
TRP	1000	6.3	8.3	7.3	82.9 ± 3.1	83.9 ± 3.7
	5000	5.4	6.3	-4.1	89.2 ± 5.5	89.1 ± 2.9
	20000	2.2	3.4	5.2	92.6 ± 5.8	92.6 ± 3.8
KYN	50	13.2	12.4	9.7	84.2 ± 9.2	92.8 ± 3.9
	250	10.4	5.1	-3.9	87.6 ± 2.7	90.2 ± 5.8
	1000	9.5	8.2	-4.1	89.1 ± 5.5	91.7 ± 3.1
KA	12.5	13.2	9.4	12.7	96.2 ± 7.5	96.7 ± 4.8
	62.5	8.3	12.7	-8.2	90.2 ± 7.6	89.9 ± 4.6
	250	6.9	10.3	13.5	92.3 ± 2.2	91.3 ± 5.4
3-HK	12.5	14.2	9.8	13.3	87.1 ± 5.2	90.5 ± 3.6
	62.5	13.7	9.6	7.1	88.4 ± 2.9	92.3 ± 5.9
	250	12.1	10.3	12.2	90.3 ± 3.8	93.4 ± 2.3
XA	12.5	10.8	8.2	10.6	91.6 ± 4.8	92.2 ± 4.5
	62.5	13.5	11.5	9.4	89.3 ± 3.1	90.7 ± 3.9
	250	11.1	13.4	8.5	91.8 ± 4.2	93.4 ± 2.2
3-HAA	12.5	13.7	11.5	7.4	88.3 ± 7.1	90.1 ± 3.9
	62.5	12.1	12.6	10.6	89.1 ± 5.9	95.0 ± 4.8
	250	10.8	9.7	-3.1	92.2 ± 3.6	91.5 ± 2.5
QA	50	9.3	10.2	7.3	89.5± 2.6	88.5± 34.2
	250	8.5	9.6	8.5	90.5± 4.6	90.1± 4.1
	1000	5.6	7.5	9.8	92.7± 3.9	93.2± 3.7

Supplemental Table 9. Stability of the analytes in plasma Trp and Kyn, KA, 3-HK, XA, 3-HAA and QA

Analytes	Concentration (ng/mL)	Room temperature for 24 h		Three freeze-thaw cycles		−80 °C for 30 days		Processed samples in 4°C for 24h	
		RE (%)	RSD (%)	RE (%)	RSD (%)	RE (%)	RSD (%)	RE (%)	RSD (%)
TRP	1000	6.5	5.1	-4.8	10.6	-6.5	9.5	8.2	9.5
	5000	-5.1	5.6	9.3	12.2	-2.3	5.2	6.2	10.4
	20000	3.6	6.9	9.6	10.7	-4.2	4.3	-3.8	11.2
KYN	50	-6.8	11.5	8.7	9.5	8.3	7.1	-4.2	8.7
	250	5.2	12.8	-3.8	8.2	-5.4	5.2	-3.9	12.5
	1000	-3.2	8.9	-5.3	7.1	-1.8	10.2	8.5	7.2
KA	12.5	-6.5	7.1	-3.8	5.3	3.6	2.7	-3.8	3.5
	62.5	-4.1	6.2	8.9	6.8	-2.5	3.6	-6.3	7.4
	250	2.2	3.3	9.4	7.5	6.4	9.2	7.2	8.2
3-HK	12.5	7.9	9.5	-2.7	10.0	9.0	10.5	-5.1	5.5
	62.5	5.7	10.0	-5.3	12.5	6.3	3.3	-9.2	7.1
	250	-2.5	9.9	7.2	10.2	8.7	7.5	-2.9	6.3
XA	12.5	6.2	10.1	4.9	13.5	-3.6	3.6	-3.3	8.2
	62.5	-3.8	12.2	5.5	13.5	-2.9	8.7	10.0	6.5
	250	4.2	13.8	8.2	11.1	7.2	8.5	-7.4	10.1
3-HAA	12.5	7.2	9.8	7.6	8.3	10.2	14.5	7.9	13.2
	62.5	5.8	8.2	6.5	5.6	8.6	13.2	11.2	10.4
	250	6.2	6.5	-3.2	-7.2	6.7	11.5	5.7	5.7
QA	50	4.8	6.4	4.1	9.5	8.2	1.9	7.3	3.2
	250	-4.1	10.8	-2.6	7.4	-4.1	5.7	-5.4	4.0
	1000	-5.4	9.8	-3.9	8.6	3.9	10.2	-7.2	9.5

Supplemental Figure Legends

Supplemental Figure 1 **Flow of the Study Population**

Supplemental Figure 2 Representative ^{1}H-NMR chromatograms of plasma derived from the control subjects and STEMI patients

(A)The PCA-X score plot.

(B)The PLS-DA score plot.

(C)The results of permutation test of control subjects and STEMI patients.

(D)The OPLS-DA score plot.

(E)The loading plot of the OPLS-DA analysis of the spectra from the control subjects and STEMI patients.

(F-G) Typical one-dimensional ^{1}H NMR spectra of serum (32 times in the vertical direction of the dotted frame).

Keys: 1, leucine; 2, 3-Hydroxybutyric acid; 3, glutamate; 4, β-glucose; 5, phenylalanine; 6, tryptophan; 7, pyroglutamate; 8, Succinate

Supplemental Figure 3 Representative chromatograms of the detection of amino acids on positive ion mode

A: Represented blank matrix, B: Represented blank matrix spiked with standard materials and IS, C: represented mixed plasma samples spiked with IS. a-3-HAA, b-XA, c-TRP, d-KA, e-AA, f-N-TRP, g-KYN, h-QA, i-3-HK.

Supplemental Figure 4 Tissue and cytological expression of IDO1

IDO1 expression profiles in the different tissues of mice indicated IDO1 expression in heart tissue.

Supplemental Figure 5 IDO1 expression was high in coronary artery plaque

To evaluate the changes in IDO1 expression in human atherosclerotic coronary arteries, we compared IDO1 expression in the coronary arteries plaque with the normal coronary arteries. The expression of IDO1 in the macrophage cells of coronary artery was identified through immunostaining of serial sections. In normal coronary vessels, there were no CD68 positive macrophage cells and the IDO1 expression was low(A). However, in the atherosclerotic coronary artery plaque, CD68+ macrophages were present and the IDO1 expression was high, compared to that in the control. Immunofluorescence staining indicated that the $CD68^{+}$ macrophages were co-localized with IDO1 in the coronary artery plaque(B).

ONLINE SUPPLEMENTAL MATERIAL REFERENCES

1. Roffi, M., Patrono, C., Collet, J.P., Mueller, C., Valgimigli, M., Andreotti, F., Bax, J.J., Borger, M.A., Brotons, C., Chew, D.P., et al. (2016). 2015 ESC Guidelines for the management of acute coronary syndromes in patients presenting without persistent ST-segment elevation: Task Force for the Management of Acute Coronary Syndromes in Patients Presenting without Persistent ST-Segment Elevation of the European Society of Cardiology (ESC). European heart journal 37, 267-315.
2. Roffi, M., Patrono, C., Collet, J.P., Mueller, C., Valgimigli, M., Andreotti, F., Bax, J.J., Borger, M.A., Brotons, C., Chew, D.P., et al. (2016). 2015 ESC Guidelines for the management of acute coronary syndromes in patients presenting without persistent ST-segment elevation: Task Force for the Management of Acute Coronary Syndromes in Patients Presenting without Persistent ST-Segment Elevation of the European Society of Cardiology (ESC). Eur Heart J 37, 267-315.
3. Amsterdam, E.A., Wenger, N.K., Brindis, R.G., Casey, D.E., Jr., Ganiats, T.G., Holmes, D.R., Jr., Jaffe, A.S., Jneid, H., Kelly, R.F., Kontos, M.C., et al. (2014). 2014 AHA/ACC Guideline for the Management of Patients with Non-ST-Elevation Acute Coronary Syndromes: a report of the American College of Cardiology/American Heart Association Task Force on Practice Guidelines. J Am Coll Cardiol 64, e139-e228.
4. Kaier, T.E., Twerenbold, R., Puelacher, C., Marjot, J., Imambaccus, N., Boeddinghaus, J., Nestelberger, T., Badertscher, P., Sabti, Z., Gimenez, M.R., et al. (2017). Direct Comparison of Cardiac Myosin-Binding Protein C With Cardiac Troponins for the Early Diagnosis of Acute Myocardial Infarction. Circulation 136, 1495-1508.
5. Tong, Q., Song, J., Yang, G., Fan, L., Xiong, W., and Fang, J. (2018). Simultaneous determination of tryptophan, kynurenine, kynurenic acid, xanthurenic acid and 5-hydroxytryptamine in human plasma by LC-MS/MS and its application to acute myocardial infarction monitoring. Biomed Chromatogr 32.
6. Cui, H., Chen, Y., Li, K., Zhan, R., Zhao, M., Xu, Y., Lin, Z., Fu, Y., He, Q., Tang, P.C., et al. (2021). Untargeted metabolomics identifies succinate as a biomarker and therapeutic target in aortic aneurysm and dissection. Eur Heart J 42, 4373-4385.
7. Zhang, L., Wei, T.T., Li, Y., Li, J., Fan, Y., Huang, F.Q., Cai, Y.Y., Ma, G., Liu, J.F., Chen, Q.Q., et al. (2018). Functional Metabolomics Characterizes a Key Role for N-Acetylneuraminic Acid in Coronary Artery Diseases. Circulation 137, 1374-1390.
8. Shetelig, C., Limalanathan, S., Hoffmann, P., Seljeflot, I., Gran, J.M., Eritsland, J., and Andersen, G.O. (2018). Association of IL-8 With Infarct Size and Clinical Outcomes in Patients With STEMI. J Am Coll Cardiol 72, 187-198.
9. Dikalova, A.E., Itani, H.A., Nazarewicz, R.R., McMaster, W.G., Flynn, C.R., Uzhachenko, R., Fessel, J.P., Gamboa, J.L., Harrison, D.G., and Dikalov, S.I. (2017). Sirt3 Impairment and SOD2 Hyperacetylation in Vascular Oxidative Stress and Hypertension. Circ Res 121, 564-

574.
10. Melhem, N.J., Chajadine, M., Gomez, I., Howangyin, K.Y., Bouvet, M., Knosp, C., Sun, Y., Rouanet, M., Laurans, L., Cazorla, O., et al. (2021). Endothelial Cell Indoleamine 2, 3-Dioxygenase 1 Alters Cardiac Function After Myocardial Infarction Through Kynurenine. Circulation 143, 566-580.
11. Chen, B., Li, H., Ou, G., Ren, L., Yang, X., and Zeng, M. (2019). Curcumin attenuates MSU crystal-induced inflammation by inhibiting the degradation of IkappaBalpha and blocking mitochondrial damage. Arthritis Res Ther 21, 193.
