# Supplementary figures and images for: Untargeted metabolomics identified kynurenine as a predictive prognostic biomarker in acute myocardial infarction
Source: Front Immunol. 2022 Nov 2;13:950441. doi: 10.3389/fimmu.2022.950441 (PMC9667794; doi:10.3389/fimmu.2022.950441)

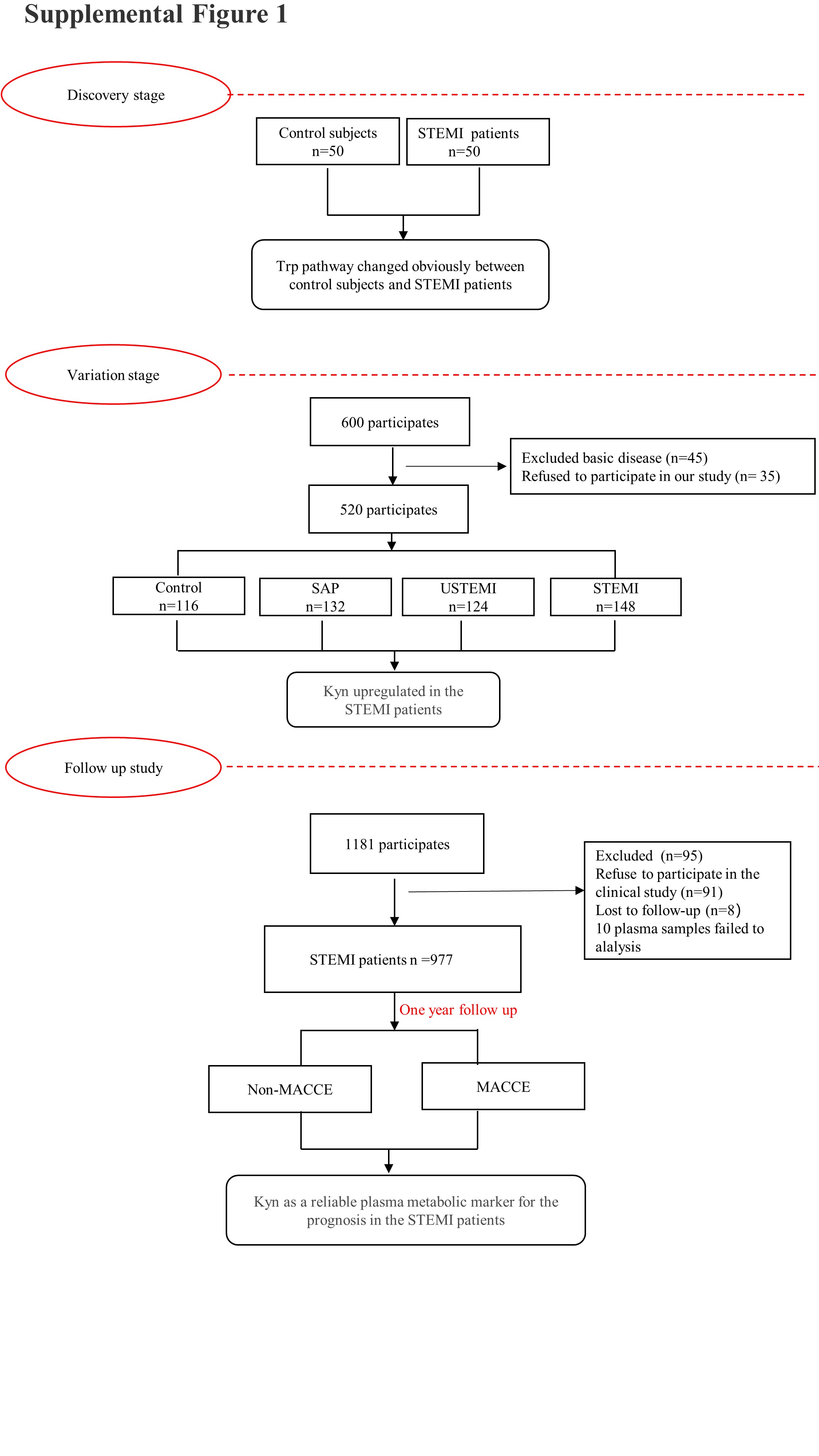

Supplement: Supplementary file 2 [file Image_1.jpg]

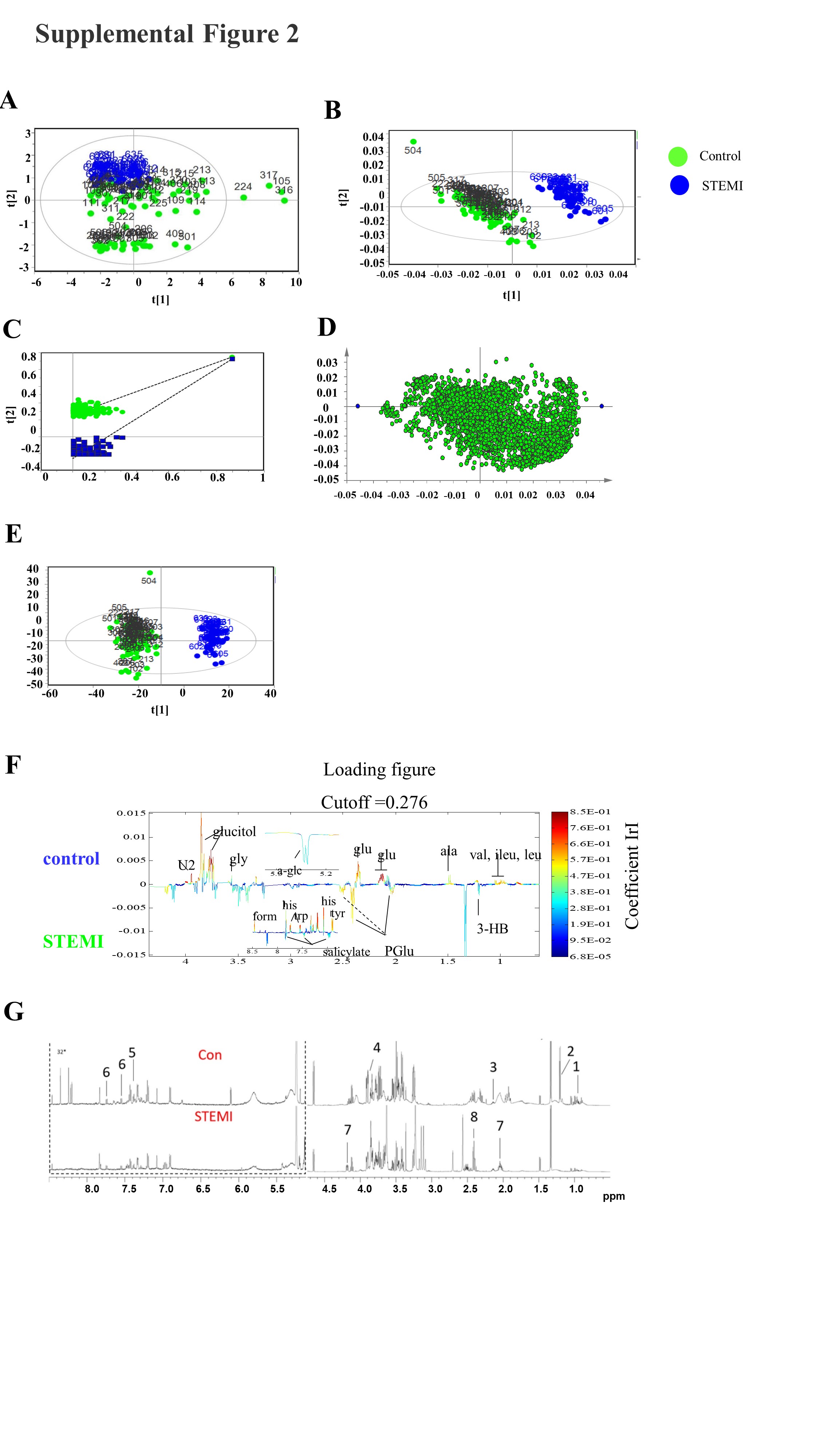

Supplement: Supplementary file 3 [file Image_2.jpg]

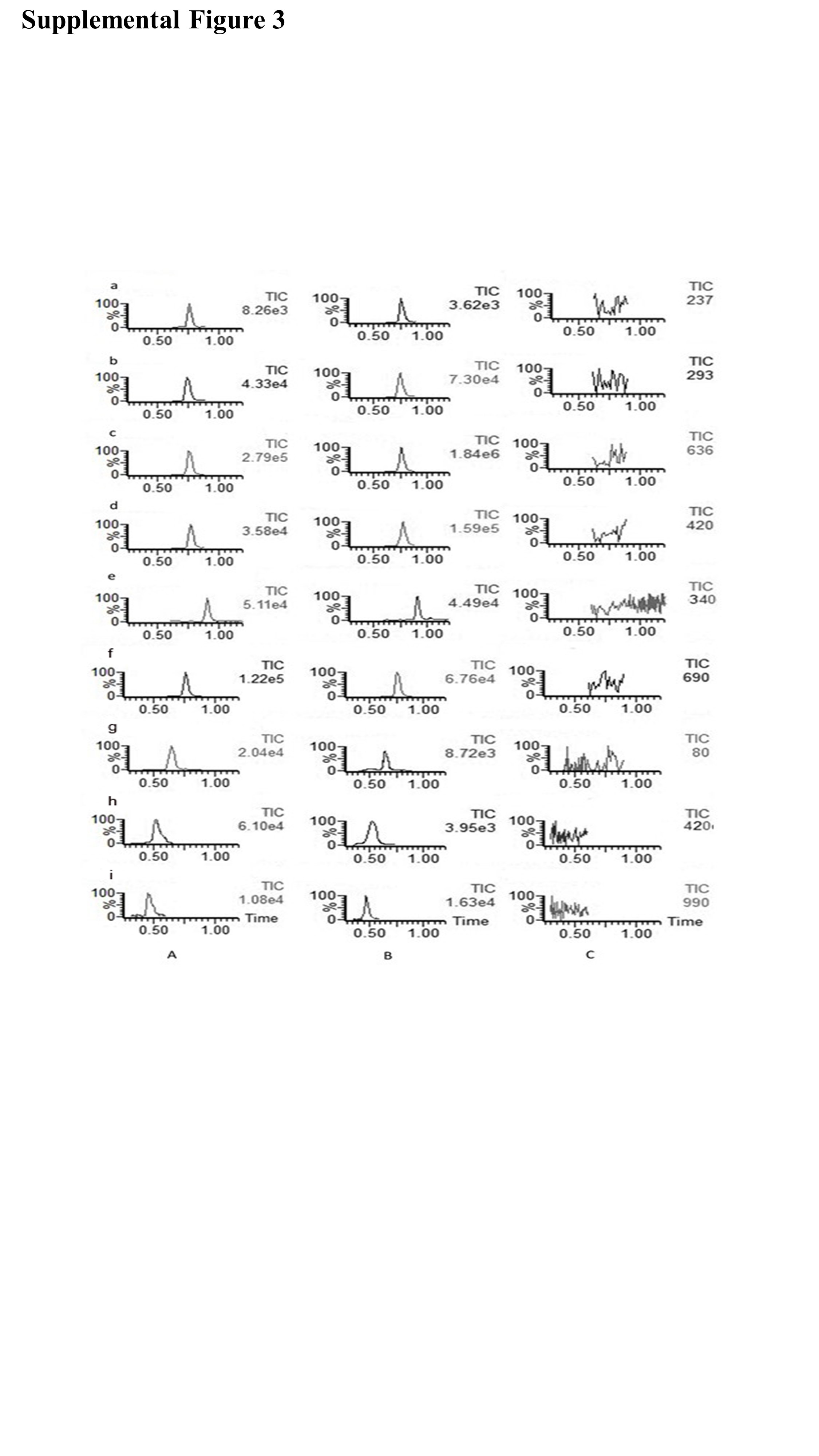

Supplement: Supplementary file 4 [file Image_3.jpg]

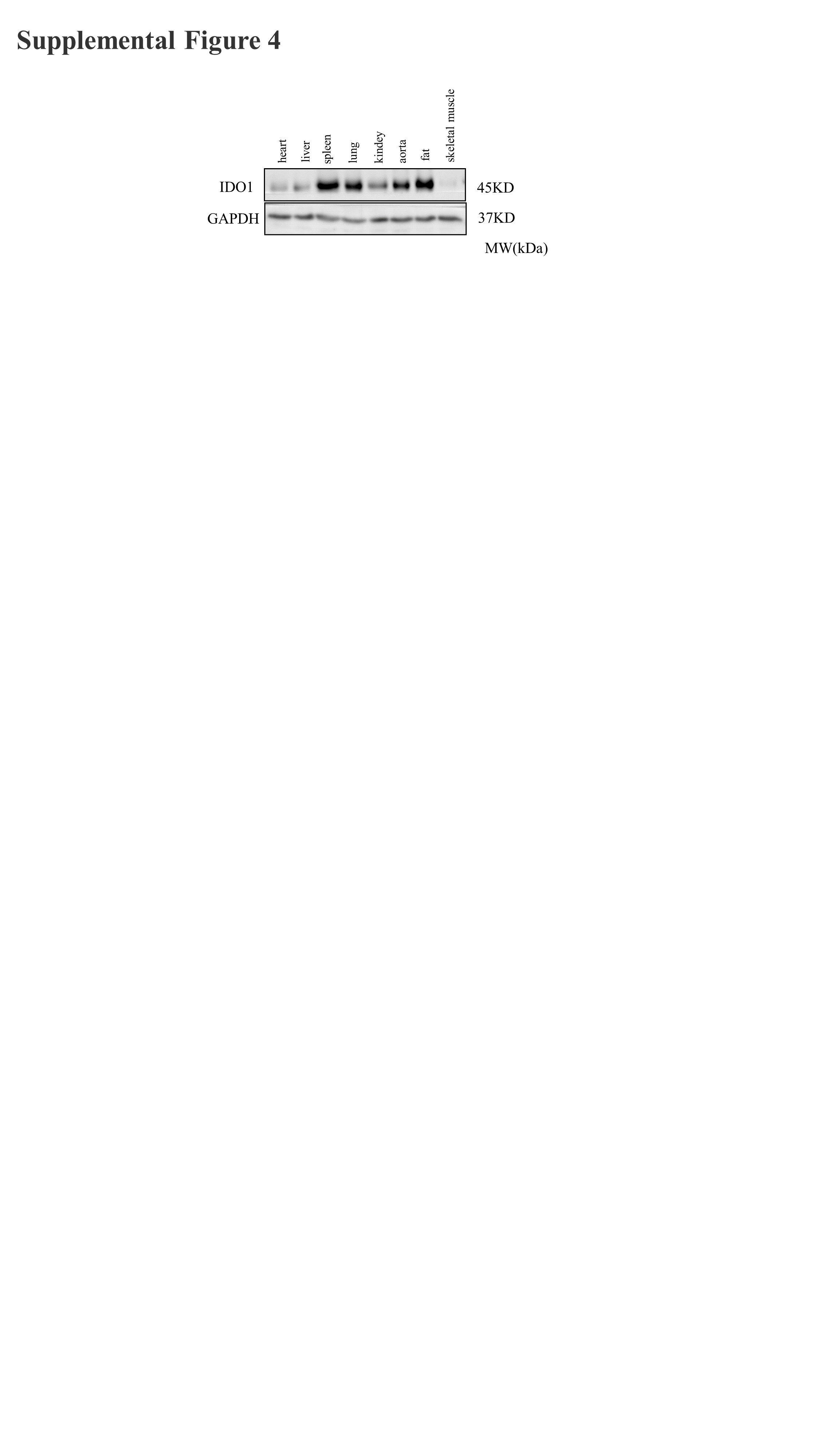

Supplement: Supplementary file 5 [file Image_4.jpg]

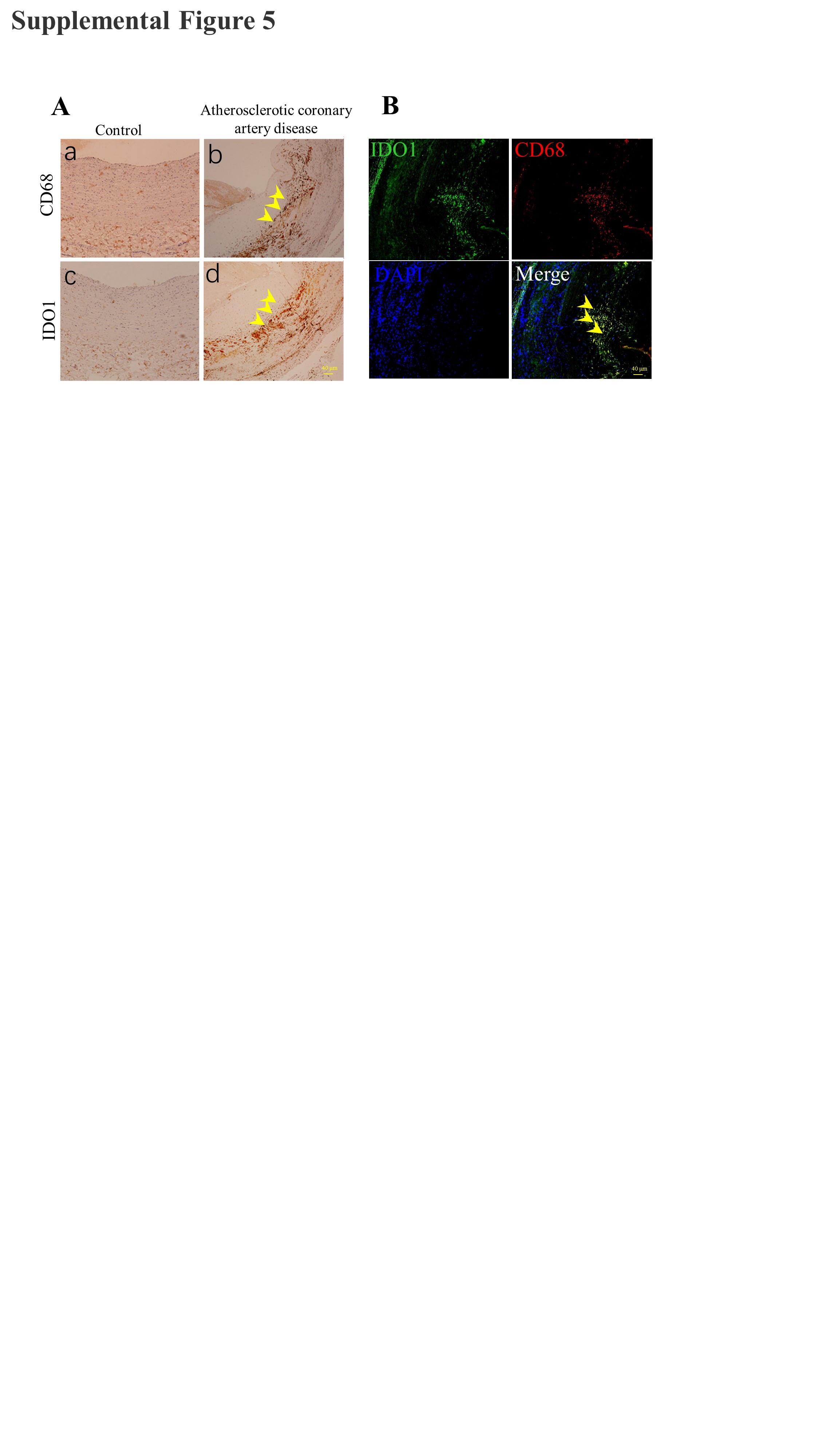

Supplement: Supplementary file 6 [file Image_5.jpg]
